# Development and validation of a new nomogram to screen for MAFLD

**DOI:** 10.1186/s12944-022-01748-1

**Published:** 2022-12-08

**Authors:** Haoxuan Zou, Fanrong Zhao, Xiuhe Lv, Xiaopu Ma, Yan Xie

**Affiliations:** Department of Gastroenterology, West China Hospital, Sichuan University, No. 37 Guoxue Alley, Chengdu, Sichuan 610041 China

**Keywords:** Metabolic dysfunction-associated fatty liver disease, LASSO, Nomogram, TyG, NHANES

## Abstract

**Background and aim:**

Metabolic dysfunction-associated fatty liver disease (MAFLD) poses significant health and economic burdens on all nations. Thus, identifying patients at risk early and managing them appropriately is essential. This study’s goal was to develop a new predictive model for MAFLD. Additionally, to improve the new model’s clinical utility, researchers limited the variables to readily available simple clinical and laboratory measures.

**Methods:**

Based on the National Health and Nutrition Examination Survey (NHANES) cycle 2017–2020.3, the study was a retrospective cross-sectional study involving 7300 participants. By least absolute shrinkage and selection operator (LASSO) regression, significant indicators independently associated with MAFLD were identified, and a predictive model called the MAFLD prediction nomogram (MPN) was developed. The study then compared the MPN with six existing predictive models for MAFLD. The model was evaluated by measuring the area under receiver operating characteristic curve (AUC), net reclassification index (NRI), integrated discrimination improvement (IDI), calibration curve, and decision curve analysis (DCA) curve.

**Results:**

In this study, researchers identified nine predictors from 33 variables, including age, race, arm circumference (AC), waist circumference (WC), body mass index (BMI), alanine aminotransferase (ALT)-to-aspartate aminotransferase (AST) ratio, triglyceride-glucose index (TyG), hypertension, and diabetes. The diagnostic accuracy of the MPN for MAFLD was significantly better than that of the other six existing models in both the training and validation cohorts (AUC 0.868, 95% confidence interval (CI) 0.858–0.877, and AUC 0.863, 95% CI 0.848–0.878, respectively). The MPN showed a higher net benefit than the other existing models.

**Conclusions:**

This nonimaging-assisted nomogram based on demographics, laboratory factors, anthropometrics, and comorbidities better predicted MAFLD than the other six existing predictive models. Using this model, the general population with MAFLD can be assessed rapidly.

**Supplementary Information:**

The online version contains supplementary material available at 10.1186/s12944-022-01748-1.

## Introduction

The high degree of heterogeneity in the etiology of nonalcoholic fatty liver disease (NAFLD) has made the current diagnostic and classification criteria for NAFLD no longer effective in guiding the clinical management of the disease and in reducing the disease burden associated with it. In this context, NAFLD has been renamed metabolism-associated fatty liver disease (MAFLD) to play an active role in guiding the individualized and precise treatment of fatty liver disease [1–4]. Inactivity, low levels of physical activity, nutritional imbalances, and unhealthy eating habits contribute to the prevalence of the disease [5]. Furthermore, MAFLD is not only closely associated with chronic hepatitis, cirrhosis, and hepatocellular carcinoma but also contributes to the progression of cardiovascular disease, chronic kidney disease, and extrahepatic malignancies in conjunction with other metabolism-related diseases, such as diabetes, hyperlipidemia and hyperuricemia [1–4].

Detecting MAFLD as early as possible to identify those who may have silent progressive fatty liver disease is crucial. Among the many diagnostic tools, the gold standard in diagnosing MAFLD is liver biopsy [6]. However, invasiveness is one of the drawbacks of liver biopsy. Ultrasonography (US), although inexpensive, depends on the experience of the operator and the sophistication of the technology. Other imaging tests, such as magnetic resonance spectroscopy (MRS), computed tomography (CT), and vibration-controlled transient elastography (VCTE), are too expensive for mass screening to be effective. Thus, there is a need to construct a simple, noninvasive, and efficient clinical prediction model capable of accurately screening MAFLD. Meanwhile, the screening tool should be widely adapted for the early detection of MAFLD in primary, secondary, and tertiary medical centers.

Previous literature describes several models based on demographics, laboratory factors, anthropometrics, and comorbidities for diagnosing NAFLD [7–11]. Among these models, the fatty liver index (FLI) has demonstrated sound diagnostic accuracy in the diagnosis of NAFLD in various populations [12, 13]. Other diagnostic models, such as the visceral adiposity index (VAI) [7], the hepatic steatosis index (HSI) [9], the ZJU index [11], and the Framingham steatosis index (FSI) [10], have also been used for NAFLD screening. Consistently, the triglyceride-glucose index (TyG), an inexpensive and reliable index for assessing insulin resistance [14], is also used to diagnose NAFLD [15]. Nevertheless, its diagnostic efficacy varies significantly between studies [11, 16, 17]. Therefore, the present study will include TyG as a variable responding to insulin resistance in the new model development and compare the new model with the above model regarding MAFLD prediction efficacy. The nomogram, a visual representation of a disease-specific prediction model based on various clinical variables, is helpful in detecting diseases early and can be easily used at all levels of medical centers [18]. Consequently, nomograms can be used to help diagnose MAFLD early. Therefore, the researchers in the present study aimed to create a novel nomogram based on demographics, laboratory factors, anthropometrics, and comorbidities to accurately detect MAFLD in the American population.

### Data source

Data were included from the National Health and Nutrition Examination Survey (NHANES), which is a nationally cross-sectional and multistage study of the nonmilitary and noninstitutionalized population of the United States. Every two years, the NHANES data are released. Each participant in the survey signed an informed consent form, and ethics review board at the National Center for Health Statistics Research has approved the protocol for this survey. In addition, this study followed the same protocol as shown in Transparent Reporting of a Multivariable Predictive Model for Individual Prognosis or Diagnosis (TRIPOD) [19].

### Participant selection

**Fig. 1:**
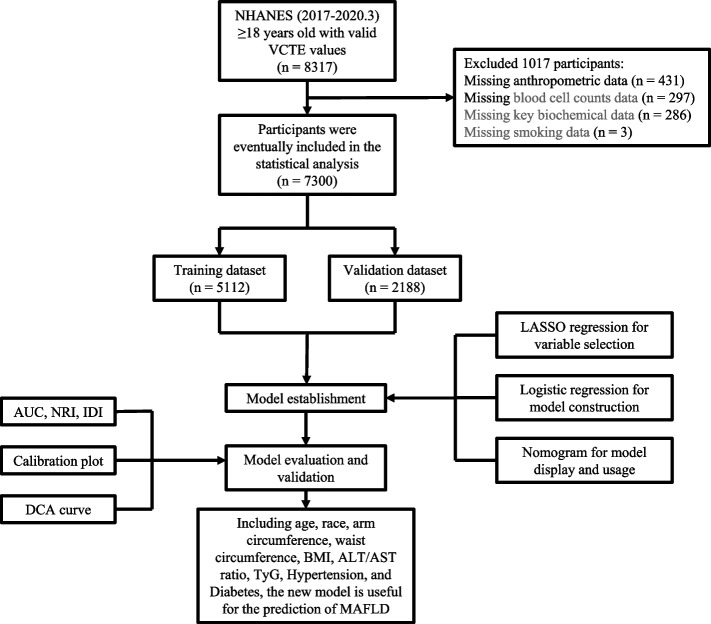
Flow diagram of study design

NHANES data (cycle 2017–2020.3) with valid vibration-controlled transient elastography (VCTE) values were used for analyses. There were 8317 subjects with valid VCTE values and ages greater than or equal to 18 years in the 2017–2020.3 NHANES database. After excluding 431 participants with no available important anthropometric data, 297 cases without key blood cell count data, 286 cases without key biochemical values, and 3 participants with no smoking data, a total of 7300 individuals were finally enrolled. An overview of the enrollment process is shown in Fig. 1.

### Demographics, Laboratory Factors, Anthropometrics, Lifestyles and Comorbidities

Data from NHANES included variables related to MAFLD from previous studies. These variables included demographics (age, sex, and race), anthropometrics (arm circumference, waist circumference, BMI, and hip circumference), lifestyles (smoking), comorbidities (hypertension and diabetes), and biomarkers such as white blood cell (WBC), hemoglobin (HB), platelets (PLT), lymphocytes (LYM), neutrophils (NEU), fasting plasma glucose (FPG), total bilirubin (TBIL), aspartate aminotransferase (AST), alanine aminotransferase (ALT), ALT to AST ratio, alkaline phosphatase (ALP), γ-glutamyl transpeptidase (GGT), triglyceride (TG), total cholesterol (TC), high-density lipoprotein cholesterol (HDL-C), total protein (TP), globulin (GLB), albumin (ALB), estimated glomerular filtration rate (eGFR), blood urea nitrogen (BUN), creatinine (CRE), and high-sensitivity C-reactive protein (hsCRP).

This study categorized race into six categories (Non-Hispanic White, Non-Hispanic Black, Other Hispanic, Mexican American, Non-Hispanic Asian, and Others) and smoking into three groups (never, former, and current). The diagnostic criteria for diabetes were FPG ≥ 7.0 mmol/L or glycohemoglobin (HbA1c) > 6.5% or random plasma glucose ≥ 11.1 mmol/L or two-hour oral glucose tolerance test (OGTT) plasma glucose ≥ 11.1 mmol/L or under anti-diabetes treatment or self-reported diabetes [20]. A systolic blood pressure (SBP) of 140 mmHg or diastolic blood pressure (DBP) of 90 mmHg or under antihypertension treatment or self-reported hypertension was used as a diagnostic criterion for hypertension [21]. The eGFR was calculated based on the chronic kidney disease epidemiology formula (CKD-EPI) [22].

### Definition of MALFD

Hepatic steatosis in this study was defined by the controlled attenuation parameter (CAP), obtained via VCTE (FibroScan®), which is a validated tool for measuring steatosis in participants with fatty liver [23]. CAP ≥ 268 dB/m was defined as significant hepatic steatosis. This cutoff value provided an AUC of 0.865 (95% CI 0.850–0.880), a sensitivity (SEN) of 0.773 (95% CI 0.690–0.838), and a specificity (SPE) of 0.812 (95% CI 0.749–0.879) [24].

A diagnosis of MAFLD was made if hepatic steatosis was present along with any of the following: overweight or obesity, diabetes, and metabolic dysfunction. At least two conditions were required for metabolic dysfunction to exist: 1) WC ≥ 102 cm in males and WC ≥ 88 cm in females, 2) hypertension, 3) hyperlipidemia (TG ≥ 1.70 mmol/L or under lipid-lowering treatment), 4) HDL-C < 1.0 mmol/L in men and < 1.3 mmol/L in women, 5) prediabetes, and 6) hsCRP > 2 mg/L [1].

### Statistical analysis

R software was used for statistical analysis (version 4.1.2). For categorical data, counts and percentages were used, and for continuous data, the mean and standard deviation (SD) were used.

For model development, in a 7:3 ratio, all 7300 participants were randomly divided into two groups for training and validation (5112 and 2188 subjects, respectively) using the “caret” package. The training dataset was used to develop the model, and internal validation was performed using the validation dataset. In addition, the researchers in this study used the “glmnet” package to perform least absolute shrinkage and selection operator (LASSO) regression. This package runs a tenfold cross-validation of the included variables before selecting the optimal lambda value. Researchers chose lambda.lse from the cross-validation results because it has the best performance but the least number of variables. Then, researchers used the “rms” package to run a logistic regression analysis. By including the variables screened in the LASSO regression, a multivariable logistic regression model was constructed. For each variable, an odds ratio (OR) and 95% confidence interval (CI) were assessed. The statistical significance levels were all two-sided. In the next step, using the “rms” package, this study developed the predictive nomogram using statistically significant variables.

For model evaluation, the receiver operating characteristic curve (ROC) operation was performed using the “pROC” package and compared against existing models. Based on Delong's method, *P* < 0.05 was considered statistically significant when comparing the area under the receiver operating characteristic curve (AUC) values. Using the AUC, the present study could distinguish true positives from false-positives based on the quality of the risk nomogram. Because the meaning of AUC increments is not intuitive, this study calculated the NRI and IDI based on the corresponding equations [25, 26].

Furthermore, researchers used the “terms” package to calculate the calibration curve and the Brier score, which was used to assess the calibration of the newly built nomogram. The decision curve analysis (DCA) curve was conducted using the “price” package, which calculates the clinical practicability of the model based on numerous threshold probabilities.

To calculate the adequate sample size, there should be at least ten outcome events per variable (EPV) when performing prediction research. Researchers expected robust estimates as the study’s sample size and outcome events were far greater than those of the EPV method [27].

## Results

### Participant characteristics

This study included 7300 subjects for analysis, and their mean age was 48.89 years. Of the 7300 participants, 3329 (45.60%) were diagnosed with MAFLD according to the diagnostic criteria mentioned above. Among the 3329 subjects with MAFLD, 1824 (54.79%) were men, and 1505 (45.21%) were women. The two sets of subjects are described in Table 1 based on their basic characteristics. At a ratio of 7:3, these participants were randomly divided into the training or validation cohort, resulting in 5112 and 2188 patients being included in each cohort. The training set participants averaged 48.81 years of age; 49.69% were men, and 2324 (35.46%) had MAFLD. A mean age of 49.10 years was observed in the validation cohort, with 49.13% being men and 1005 (45.93%) having MAFLD. For baseline characteristics, there were no significant differences between the two datasets (as shown in Table S1).

**Table 1:** Baseline characteristics of participants

	Overall (*n* = 7300)	Non-MAFLD (*n* = 3971)	MAFLD (*n* = 3329)	*P* value
Age (years)	48.89 ± 18.06	46.18 ± 18.87	52.13 ± 16.47	< 0.001
Sex, n (%)				< 0.001
Female	3685 (50.48)	2180 (54.90)	1505 (45.21)	
Male	3615 (49.52)	1791 (45.10)	1824 (54.79)	
Race, n (%)				< 0.001
Non-Hispanic Black	1817 (24.89)	1113 (28.03)	704 (21.15)	
Non-Hispanic White	2563 (35.11)	1343 (33.82)	1220 (36.65)	
Other Hispanic	763 (10.45)	405 (10.20)	358 (10.75)	
Non-Hispanic Asian	884 (12.11)	544 (13.70)	340 (10.21)	
Mexican American	918 (12.58)	367 (9.24)	551 (16.55)	
Other	355 (4.86)	199 (5.01)	156 (4.69)	
AC (cm)	33.54 ± 5.24	31.39 ± 4.47	36.11 ± 4.92	< 0.001
HC (cm)	106.86 ± 14.15	101.54 ± 11.50	113.22 ± 14.40	< 0.001
WC (cm)	100.07 ± 16.96	91.66 ± 13.79	110.09 ± 14.79	< 0.001
BMI (kg/m^2^)	29.63 ± 7.05	26.48 ± 5.52	33.40 ± 6.84	< 0.001
WBC (× 10^9^/l)	7.21 ± 5.14	6.92 ± 6.67	7.55 ± 2.16	< 0.001
LYM (× 10^9^/l)	2.25 ± 4.33	2.21 ± 5.81	2.31 ± 0.88	0.314
NEU (× 10^9^/l)	4.15 ± 1.69	3.94 ± 1.69	4.39 ± 1.64	< 0.001
HB (g/l)	14.03 ± 1.54	13.86 ± 1.51	14.24 ± 1.55	< 0.001
PLT (× 10^9^/l)	246.47 ± 64.89	243.89 ± 63.93	249.55 ± 65.89	< 0.001
FPG (mmol/l)	5.62 ± 1.95	5.22 ± 1.36	6.10 ± 2.39	< 0.001
TG (mmol/l)	1.54 ± 1.12	1.24 ± 0.79	1.91 ± 1.34	< 0.001
TyG	8.64 ± 0.65	8.40 ± 0.55	8.93 ± 0.65	< 0.001
ALT (u/l)	22.32 ± 18.99	18.97 ± 18.59	26.32 ± 18.69	< 0.001
AST (u/l)	21.89 ± 14.42	21.11 ± 15.15	22.82 ± 13.44	< 0.001
ALT/AST ratio	0.99 ± 0.36	0.88 ± 0.29	1.13 ± 0.38	< 0.001
TC (mmol/l)	4.79 ± 1.05	4.72 ± 1.03	4.87 ± 1.06	< 0.001
HDL-C (mmol/l)	1.38 ± 0.40	1.48 ± 0.41	1.25 ± 0.36	< 0.001
GGT (u/l)	31.60 ± 51.68	26.59 ± 54.93	37.59 ± 46.81	< 0.001
ALP (u/l)	77.71 ± 25.80	74.75 ± 25.81	81.23 ± 25.33	< 0.001
ALB (g/l)	40.79 ± 3.34	41.08 ± 3.37	40.44 ± 3.28	< 0.001
GLB (g/l)	30.88 ± 4.32	30.60 ± 4.35	31.21 ± 4.25	< 0.001
TP (g/l)	71.67 ± 4.42	71.68 ± 4.49	71.66 ± 4.34	0.796
TBIL (mg/dl)	7.85 ± 4.74	8.06 ± 5.04	7.60 ± 4.35	< 0.001
hsCRP (mg/l)	3.92 ± 8.30	3.03 ± 7.19	4.98 ± 9.34	< 0.001
CRE (mg/dl)	79.03 ± 39.21	78.34 ± 36.05	79.86 ± 42.67	0.100
BUN (mg/dl)	5.25 ± 2.02	5.12 ± 1.91	5.40 ± 2.13	< 0.001
eGFR (mg/min/1.73m^2^)	95.59 ± 23.59	97.60 ± 23.75	93.20 ± 23.18	< 0.001
Hypertension, n (%)				< 0.001
No	4662 (63.86)	2889 (72.75)	1773 (53.26)	
Yes	2638 (36.14)	1082 (27.25)	1556 (46.74)	
Diabetes, n (%)				< 0.001
No	5895 (80.75)	3574 (90.00)	2321 (69.72)	
Yes	1405 (19.25)	397 (10.00)	1008 (30.28)	
Smoking, n (%)				< 0.001
Never	4367 (59.82)	2469 (62.18)	1898 (57.01)	
Former	1654 (22.66)	750 (18.89)	904 (27.16)	
Current	1279 (17.52)	752 (18.94)	527 (15.83)	
FLI	55.25 ± 32.65	37.28 ± 28.96	76.68 ± 22.19	< 0.001
HSI	38.97 ± 8.52	34.82 ± 6.60	43.92 ± 7.88	< 0.001
VAI	2.17 ± 2.26	1.58 ± 1.61	2.88 ± 2.67	< 0.001
FSI	-0.89 ± 1.87	-1.88 ± 1.42	0.28 ± 1.66	< 0.001
ZJU	40.79 ± 8.34	36.68 ± 6.38	45.69 ± 7.73	< 0.001

Data are presented as means ± SD and absolute or relative percentages

### Selection of main predictors of MAFLD

Because LASSO regression cannot handle unordered multicategorical variables, this study split race and smoking into several multiple dichotomous variables before performing LASSO regression. If LASSO regression screened out any of the split variables, the present study included the original unordered multicategorical variables for that variable in the multivariate logistic regression to construct the model.

**Fig. 2:**
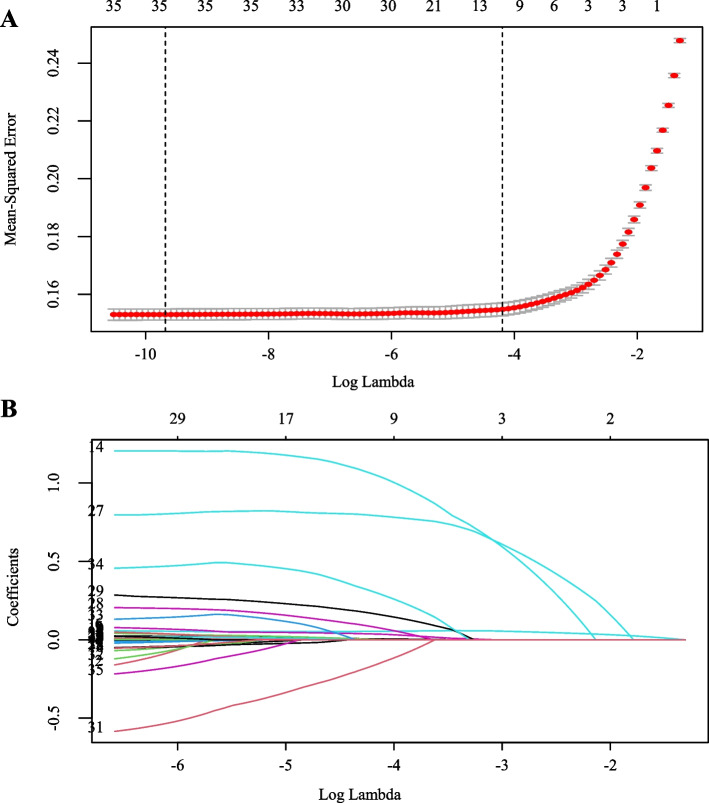
Variables selection using the LASSO regression. **A** Selection of the tuning parameter lambda in the LASSO model via tenfold cross-validation based on minimum criteria. Mean-squared error (MSE) from the LASSO regression cross-validation procedure was plotted as a function of log lambda. The y-axis indicates the MSE. The x-axis indicates the log lambda. Numbers along the upper x-axis represent the average number of predictors. Red dots indicate average MSE values for each model with a given lambda, and vertical bars through the red dots show the upper and lower values of the MSE. The vertical black lines define the optimal values of lambda, where the model provides its best fit to the data based on 1 standard error criteria. The optimal lambda value of 0.014 with log lambda = -4.269 was selected. **B** The LASSO coefficient profiles of clinical features. The dotted vertical line was plotted at the value selected using tenfold cross-validation in A. The nine resulting variables with non-zero coefficients are indicated in the plot

Among the 33 demographics, laboratory factors, anthropometrics, lifestyles, and comorbidities, nine significant predictors of MAFLD were selected in the training set (as shown in Fig. 2A, B). These predictors were age, race (Non-Hispanic Black and Mexican American), arm circumference, waist circumference, BMI, ALT/AST ratio, TyG, hypertension, and diabetes.

### Construction of a new predictive model of MAFLD

**Fig. 3:**
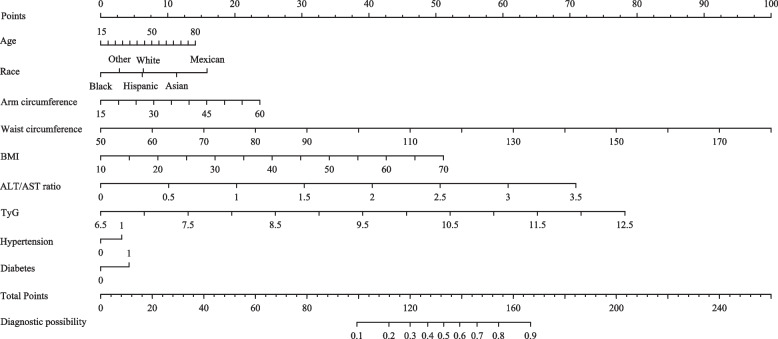
The nomogram (the MPN) represents the predicted probability of MAFLD on a scale of 0 to 260. For each predictor, draw a vertical line straight up to the point axis and note the corresponding points. Sum the points from each predictor, and the total score corresponding to a predicted probability of major postoperative complications can be found at the bottom of the nomogram. Hypertension: 0 means no hypertension, 1 means hypertension; Diabetes: 0 means no diabetes, 1 means diabetes; Race: White means Non-Hispanic White, Black means Non-Hispanic Black, Asian means Non-Hispanic Asian, Mexican means Mexican American, and others mean other race (including multi-racial)

After LASSO regression analysis, nine of the original 33 variables were selected as optimal variables. Then, to create a new predictive model, this study used multivariate logistic regression. Table S2 shows the results of the logistic regression analysis of these nine variables, and all nine predictors were statistically significant. This set of predictors, which were independent of each other, was combined to develop a nomogram that quantified MAFLD risk (as shown in Fig. 3). In addition, in order to make this nomogram more convenient to use, we have made it dynamic and searchable directly on the Internet (https://themafldpredicitonmodel.shinyapps.io/DynamicMPN/) (as shown in Figure S1).

### Performance of the MPN in AUC, reclassification, and calibration curve

**Fig. 4:**
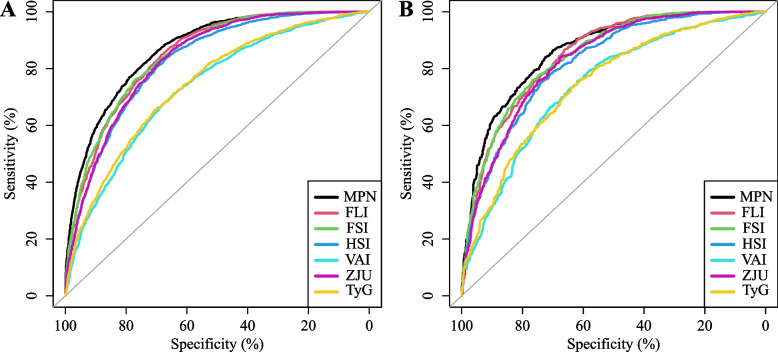
ROC curves for predicting MAFLD in the training dataset (**A**) and the validation dataset (**B**). The x-axis is the specificity; the y-axis is the sensitivity

Several different prediction models were compared based on calculating their specificity and sensitivity, including the MPN, FLI, HSI, VAI, FSI, ZJU, and TyG. Figure 4A, B shows the ROC curves of the MPN and the other six models in the training and validation cohorts. The performance details are shown in Tables 2 and 3. Compared with FLI (0.849, 95% CI 0.838–0.859), HSI (0.825, 95% CI 0.814–0.836), VAI (0.732, 95% CI 0.718–0.746), FSI (0.848, 95% CI 0.838–0.859), ZJU (0. 833, 95% CI 0.822–0.844), and TyG (0.743, 95% CI 0.729–0. 756), MPN had the highest AUC value for predicting MAFLD risk in the training dataset (0.868, 95% CI 0.858–0.877), and the MPN’s AUC values were significantly different from those of the six models described above (all *P* < 0.001). There was 86.3% sensitivity, 69.7% specificity, 70.4% PPV and 85.9% NPV of the MPN, while the cutoff value was 0.360. In addition, in the validation dataset, compared with the FLI (0.847, 95% CI 0.832–0.863), HSI (0.818, 95% CI 0.801–0.836), VAI (0.740, 95% CI 0.719–0.761), FSI (0.846, 95% CI 0.830–0.862), ZJU (0.829, 95% CI 0.812–0.845), and TyG (0.744, 95% CI 0.723–0.764), the MPN also had the highest AUC value for predicting the risk of MAFLD (0.863, 95% CI 0.848–0.878). Similarly, in the validation set, statistically significant differences were found between the MPN and the above models’ AUC values (all *P* < 0.001). There was 84.0% sensitivity, 72.5% specificity, 72.2% PPV and 84.2% NPV of the MPN, while the cutoff value was 0.397. Separate ROC curves and formulae for the above models can be found in Figures S1,2,3,4,5,6,7. In further subgroup analyses, the AUC of MPN remained higher in men and women, under and over 60 years of age, with and without hypertension, and with and without diabetes than in other existing models (shown in Tables S3,4,5,6,7,8,9,10, Figures S9,10,11,12).

**Table 2:** Performance assessment of the MPN and other scoring methods for the prediction of MAFLD in the training cohort

Models	AUC (95% CI)	*P*^1^values	SEN (95% CI)	SPE (95% CI)	PPV (95% CI)	NPV (95% CI)	Cutoff values	NRI (95% CI)	*P*^2^values	IDI (95% CI)	*P*^3^values
MPN	0.868 (0.858–0.877)	-	0.863 (0.849–0.887)	0.697 (0.680–0.714)	0.704 (0.687–0.721)	0.859 (0.845–0.874)	0.360	Ref	-	Ref	-
FLI	0.849 (0.838–0.859)	< 0.001	0.846 (0.832–0.861)	0.686 (0.669–0.703)	0.692 (0.675–0.709)	0.843 (0.828–0.858)	49.652	0.027 (0.009–0.045)	0.004	0.030 (0.007–0.053)	0.012
HSI	0.825 (0.814–0.836)	< 0.001	0.836 (0.821–0.852)	0.669 (0.651–0.686)	0.678 (0.661–0.695)	0.831 (0.815–0.846)	36.558	0.055 (0.034–0.076)	< 0.001	0.058 (0.033–0.083)	< 0.001
VAI	0.732 (0.718–0.746)	< 0.001	0.711 (0.692–0.729)	0.645 (0.627–0.662)	0.625 (0.607–0.644)	0.728 (0.710–0.745)	1.541	0.204 (0.176–0.232)	< 0.001	0.189 (0.163–0.215)	< 0.001
FSI	0.848 (0.838–0.859)	< 0.001	0.762 (0.744–0.779)	0.770 (0.754–0.786)	0.734 (0.717–0.752)	0.795 (0.780–0.810)	-0.943	0.029 (0.009–0.049)	0.004	0.034 (0.010–0.058)	0.005
ZJU	0.833 (0.822–0.844)	< 0.001	0.829 (0.814–0.844)	0.685 (0.668–0.703)	0.687 (0.670–0.704)	0.828 (0.813–0.843)	38.727	0.045 (0.024–0.066)	< 0.001	0.050 (0.025–0.075)	< 0.001
TyG	0.743 (0.729–0.756)	< 0.001	0.650 (0.636–0.674)	0.706 (0.689–0.722)	0.650 (0.630–0.669)	0.710 (0.693–0.727)	8.640	0.200 (0.171–0.229)	< 0.001	0.185 (0.159–0.211)	< 0.001

^1^*P* values for the difference of AUC between the MPN and other models

^2^*P* values for the difference of NRI between the MPN and other models

^3^*P* values for the difference of IDI between the MPN and other models

**Table 3:** Performance assessment of the MPN and other scoring methods for the prediction of MAFLD in the validation cohort

Models	AUC (95% CI)	*P*^1^values	SEN (95% CI)	SPE (95% CI)	PPV (95% CI)	NPV (95% CI)	Cutoff values	NRI (95% CI)	*P*^2^values	IDI (95% CI)	*P*^3^values
MPN	0.863 (0.848–0.878)	-	0.840 (0.817–0.862)	0.725 (0.700–0.751)	0.722 (0.696–0.748)	0.842 (0.820–0.864)	0.397	Ref	-	Ref	-
FLI	0.847 (0.832–0.863)	< 0.001	0.869 (0.848–0.890)	0.659 (0.632–0.686)	0.684 (0.659–0.710)	0.855 (0.832–0.878)	48.750	0.036 (0.008–0.064)	0.012	0.033 (-0.002–0.068)	0.071
HSI	0.818 (0.801–0.836)	< 0.001	0.751 (0.725–0.778)	0.739 (0.714–0.764)	0.710 (0.682–0.737)	0.778 (0.753–0.802)	38.513	0.074 (0.041–0.107)	< 0.001	0.079 (0.041–0.117)	< 0.001
VAI	0.740 (0.719–0.761)	< 0.001	0.756 (0.730–0.783)	0.619 (0.591–0.646)	0.628 (0.600–0.655)	0.749 (0.722–0.776)	1.393	0.190 (0.148–0.232)	< 0.001	0.177 (0.136–0.218)	< 0.001
FSI	0.846 (0.830–0.862)	< 0.001	0.759 (0.733–0.786)	0.768 (0.743–0.792)	0.735 (0.708–0.762)	0.790 (0.766–0.813)	-0.974	0.037 (0.008–0.065)	0.011	0.041 (0.005–0.077)	0.024
ZJU	0.829 (0.812–0.845)	< 0.001	0.842 (0.819–0.864)	0.672 (0.645–0.699)	0.686 (0.660–0.711)	0.833 (0.810–0.857)	38.502	0.050 (0.018–0.082)	0.002	0.051 (0.013–0.089)	0.008
TyG	0.744 (0.723–0.764)	< 0.001	0.732 (0.705–0.760)	0.641 (0.613–0.668)	0.634 (0.606–0.662)	0.738 (0.711–0.765)	8.514	0.191 (0.149–0.233)	< 0.001	0.179 (0.139–0.219)	< 0.001

^1^*P* values for the difference of AUC between the MPN and other models, ^2^*P* values for the difference of NRI between the MPN and other models, ^3^*P* values for the difference of IDI between the MPN and other models

Because the meaning of AUC increments is not intuitive, to further evaluate the recognition ability of the MPN, this study calculated the NRI and IDI and compared them with other models. Compared with the FLI, HSI, VAI, FSI, ZJU, TyG, NRI and IDI values of the MPN were higher than those of the existing models in both the training and validation cohorts (as shown in Tables 2 and 3). These results demonstrate that the MPN has better discriminatory power and outperforms these commonly used predictive models.

**Fig. 5:**
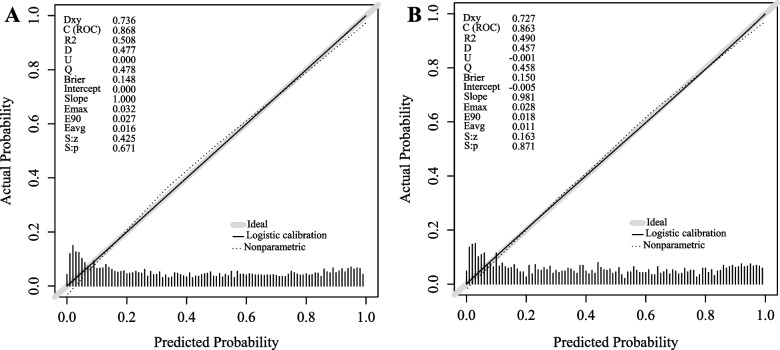
The performance of the new nomogram was assessed by calibration curves in the training dataset (**A**) and the validation dataset (**B**). The x-axis is the nomogram predicted probability of MAFLD; the y-axis is actual probability

Consistently, the calibration curve of the MPN for screening MAFLD showed good consistency between both the training set (*P* = 0.671, Brier score = 0.148) and the validation set (*P* = 0.871, Brier score = 0.150) (as shown in Fig. 5A, B). A calibration curve that is as close to the diagonal line as possible (*P* > 0.05, Brier score < 0.25) indicates a more accurate prediction.

### Clinical utility evaluation of the MPN

**Fig. 6:**
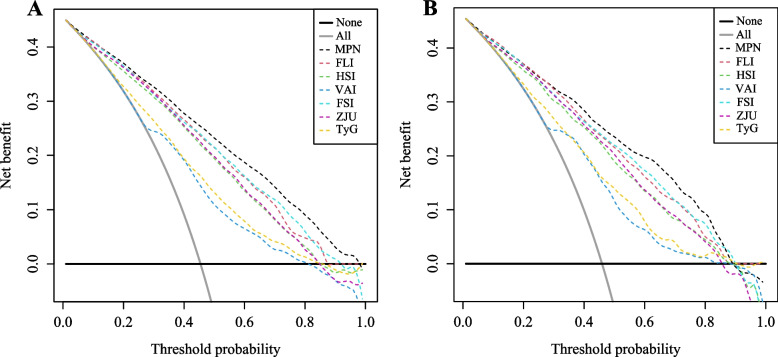
The clinical utility of the nomogram was evaluated by decision curves in the training dataset (**A**) and the validation dataset (**B**). The x-axis measures the threshold probability. The y-axis represents net benefits, calculated by subtracting the relative harms (false positives) from the benefits (true positives)

By quantifying the net benefit probabilities at the 0.0 to 1.0 threshold, this study performed a DCA to assess the clinical usefulness of the MPN and compare it with other existing models. Simply put, there is a direct correlation between the decision curve and the net benefit of the model's clinical decisions based on the distance from the two extreme curves: clinical decisions made by the model are more beneficial when they are farther from the two extreme curves. As shown in Fig. 6A, B, the MPN showed a higher net benefit than the other six models (the FLI, the HSI, the AVI, the FSI, the ZJU, and the TyG) in both the training and validation sets. Based on these results, MPN is comparable to VCTE in diagnosing MAFLD. This newly built nomogram does not require imaging or liver biopsy to identify hepatic steatohepatitis. This screening tool can be used to determine if an individual needs further, more accurate testing to confirm MAFLD diagnosis.

## Discussion

Hepatic steatosis is the basis of diagnosing MAFLD [1]. However, tests such as US, VCTE, CT, MRS, and biopsy that can respond to hepatic steatosis are not suitable for mass screening. Because MAFLD lacks specific clinical symptoms, clinicians often find MAFLD accidentally while performing tests for other diseases or during annual physical examinations. Unlike imaging or liver biopsy tests, demographics (age, race, etc.), laboratory factors (ALT, AST, TG, FPG, etc.), anthropometrics (AC, WC, BMI, etc.), and comorbidities (diabetes, hypertension, etc.) are routinely monitored during annual physical examinations and are easily available at primary, secondary, and tertiary medical centers. Therefore, developing a simple and practical diagnostic tool based on these easily accessible indicators appears significantly essential in resolving this problem.

In the present study, researchers used a large sample in the Americas with standardized demographic, anthropometric, laboratory, and comorbidity measures. Additionally, when constructing the model for predicting MAFLD, to enhance the clinical usefulness of the newly built nomogram, the candidate variables were limited to simple clinical and laboratory measures that are readily available. Therefore, based on data for 7300 participants from the NHANES database, this study developed a new clinical prediction model for the early screening of MAFLD, which includes nine indicators, such as age, race, arm circumference, waist circumference, BMI, ALT/AST ratio, TyG, hypertension, and diabetes. Further examinations, such as imaging tests or liver biopsy, were recommended for eligible subjects with a high probability of MAFLD. Using this novel approach, clinical workers can quickly and accurately identify subjects potentially at risk for MAFLD.

The present study innovatively included TyG as an index of response to insulin resistance in the variable screening. Due to its strong correlation with MALFD in this research, TyG was included in the final model construction. Insulin resistance is an integral and central part of the development of MAFLD [28]. At the beginning, the TyG was invented as a reliable alternative indicator of the response to insulin resistance [14, 29, 30]. This index is new and reliable for assessing insulin resistance involving fasting glucose and triglycerides, with the advantages of being inexpensive and easy to use [31, 32]. A close association between the TyG and patients with NAFLD has been confirmed [15, 17, 33]. Some studies have also used the TyG as a diagnostic model for NAFLD, and its diagnostic efficacy varies widely among articles [11, 15–17]. However, the results of most articles showed that the predictive value of the TyG for NAFLD was not satisfactory. The diagnostic value of the TyG index for the risk of MAFLD in this study was also lower than that of models such as the FLI, the HSI, the FSI, and the ZJU. Moreover, the MPN showed better predictive efficacy than the TyG alone in both the training and validation datasets, implying that TyG needs to be combined with other predictors to diagnose MAFLD more accurately.

Although there are many predictive models for the diagnosis of NAFLD in previous studies, these models are based on logistic regression for variable screening and model construction, and they suffer from a small sample size, lack of complete model evaluation methods, lack of internal or external validation, and lack of comparison with existing models. In addition, although patients diagnosed with MALFD and NAFLD mostly overlapped, the populations of MAFLD and NAFLD were not exactly the same. A recent retrospective study using NHANES data showed that MAFLD criteria incorporated a significant proportion of patients not identified by NALFD criteria and that the MAFLD patient cohort had more comorbidities and a worse prognosis than patients with NAFLD only [34]. Therefore, there is a need to construct an early diagnosis model for MALFD to facilitate early intervention in this population. In contrast to previous studies, this study randomly divided the study population into training and validation cohorts, with the training set used for model development and the validation set used for internal validation of the model. LASSO regression analysis was used to screen indicators, and multivariate logistic regression analysis was also performed to structure the MPN. Additionally, the newly built model was shown as a nomogram (normal and dynamic nomograms), which can be easily used on a web page or electronically on a computer or printed out directly for clinical use in paper form. Regarding model evaluation, AUC, NRI, IDI, calibration curve, and DCA curve were systematically evaluated to verify the accuracy and stability of this study’s model. In addition, the new model was compared with the FLI [8], HSI [9], VAI [7], ZJU [11], FSI [10], and TyG [15] regarding the AUC, NRI, IDI, and DCA curves. Even in further subgroup analyses, the AUC of MPN remained superior to other models (as shown in Tables S3,4,5,6,7,8,9,10 Figures S8,9,10,11,12), however, the optimal cutoff values were not the same for each subgroup, so the predicted and observed risk of MAFLD may be slightly different, depending on patient characteristics (men versus women, under 60 years of age versus over 60 years of age, non-hypertension versus hypertension, non-diabetes versus diabetes). In summary, the newly built model outperforms the other six existing models in all aspects, indicating that its predictive efficacy is greater than the others overall (as shown in Figs. 4, 6 and Table 2).

### Strength and limitations

Several advantages of this study are worth mentioning. First, in this study, hepatic steatosis was measured by VCTE, which is more sensitive and accurate than US [35]. Second, this study used a large sample of 7730 participants from the NHANES database to make the results reliable. Third, in developing the model for predicting MALFD, researchers restricted the variables to simple and available measures to improve the clinical utility of this study’s nomogram. However, researchers acknowledge that there are several limitations. First, in NHANES, participants representing the noninstitutionalized civilian population in the Americas were selected through a multistage, probability sampling design and were not chosen through random sampling. Therefore, when analyzing NHANES data, sample weights that allow the findings to reflect the American population-wide characteristics should be considered. This study did not perform a weighted analysis, and samples with missing data were directly excluded, so the baseline characteristics of the population in this study are not representative of the actual situation in the Americas. However, the prediction model only needs to predict the sampled participants, and the unweighting does not affect the accuracy of the prediction model constructed in this study. Second, although VCTE is more accurate than ultrasound for diagnosing hepatic steatosis, it is still inferior to liver biopsy. Thus, this nomogram may potentially underdetect or underdiagnose MAFLD. Third, although this study divided whole sets into the training and validation sets at the beginning and used the validation set for internal validation, this study still lacks external validation to further evaluate the model efficacy. To verify the findings externally, multicenter studies that include participants from different regions are needed.

## Conclusion

Compared with other existing models, this study developed an effective clinical nomogram, called the MPN, for screening MAFLD in a large US population. Depending on the assessment of risk, clinicians can develop individualized treatment plans for subjects. For individuals at high risk of MAFLD, additional diagnostic tests should be ordered, which will allow early lifestyle or medical intervention and prevent further progression of the disease. Thus, the prediction model constructed in this study for MAFLD has significant clinical value. However, the MPN still needs external validation in other cohorts to prove its generalizability.

## Supplementary Information

**Additional file 1.** Supplementary figures

**Additional file 2.** Supplementary tables

## Data Availability

Data were included from the NHANES (https://www.cdc.gov/nchs/nhanes/index.htm).

## Abbreviations

NHANES: National health and nutrition examination survey
TRIPOD: Transparent reporting of a multivariable prediction model for individual prognosis or diagnosis
MAFLD: Metabolic dysfunction-associated fatty liver disease
NAFLD: Nonalcoholic fatty liver disease
BMI: Body mass index
AC: Arm circumference
WC: Waist circumference
HC: Hip circumference
WBC: White blood cell
NEU: Neutrophils
LYM: Lymphocytes
HB: Hemoglobin
PLT: Platelets
TG: Triglyceride
FPB: Fasting plasma glucose
HbA1c: Glycohemoglobin
OGTT: Oral glucose tolerance test
ALT: Alanine aminotransferase
AST: Aspartate aminotransferase
GGT: γ- Glutamyl transpeptidase
ALP: Alkaline phosphatase
HDL-C: High-density lipoprotein cholesterol
TC: Total lipoprotein cholesterol
ALB: Albumin
GLB: Globulin
TP: Total protein
TBIL: Total bilirubin
hsCRP: High-sensitivity C-reactive protein
BUN: Blood urea nitrogen
CRE: Creatinine
eGFR: Estimated glomerular filtration rate
SBP: Systolic blood pressure
DBP: Diastolic blood pressure
CKD-EPI: Chronic kidney disease epidemiology equation
VCTE: Vibration-controlled transient elastography
CT: Computed tomography
MRS: Magnetic resonance spectroscopy
US: Ultrasonography
CAP: Controlled attenuation parameter
OR: Odds ratio
SD: Standard deviation
CI: Confidence interval
EPV: Events per variable
LASSO: Least absolute shrinkage and selection operator
MSE: Mean-squared error
ROC: Receiver operating characteristic curve
SEN: Sensitivity
SPE: Specificity
PPV: Positive predictive value
NPV: Negative predictive value
AUC: Area under receiver operating characteristic curve
NRI: Net reclassification index
IDI: Integrated discrimination improvement
DCA: Decision curve analysis
FLI: Fatty liver index
FSI: Framingham steatosis index
HSI: Hepatic steatosis index
VAI: Visceral Adiposity Index
TyG: Triglyceride-glucose
MPN: MAFLD prediction nomogram
