# Supplementary material for: Development and validation of a new nomogram to screen for MAFLD
Source: Lipids Health Dis. 2022 Dec 8;21:133. doi: 10.1186/s12944-022-01748-1 (PMC9730620; doi:10.1186/s12944-022-01748-1)
Supplement: Supplementary file 1 — Additional file 1. Supplementary figures [file 12944_2022_1748_MOESM1_ESM.pptx]

## Slide 1
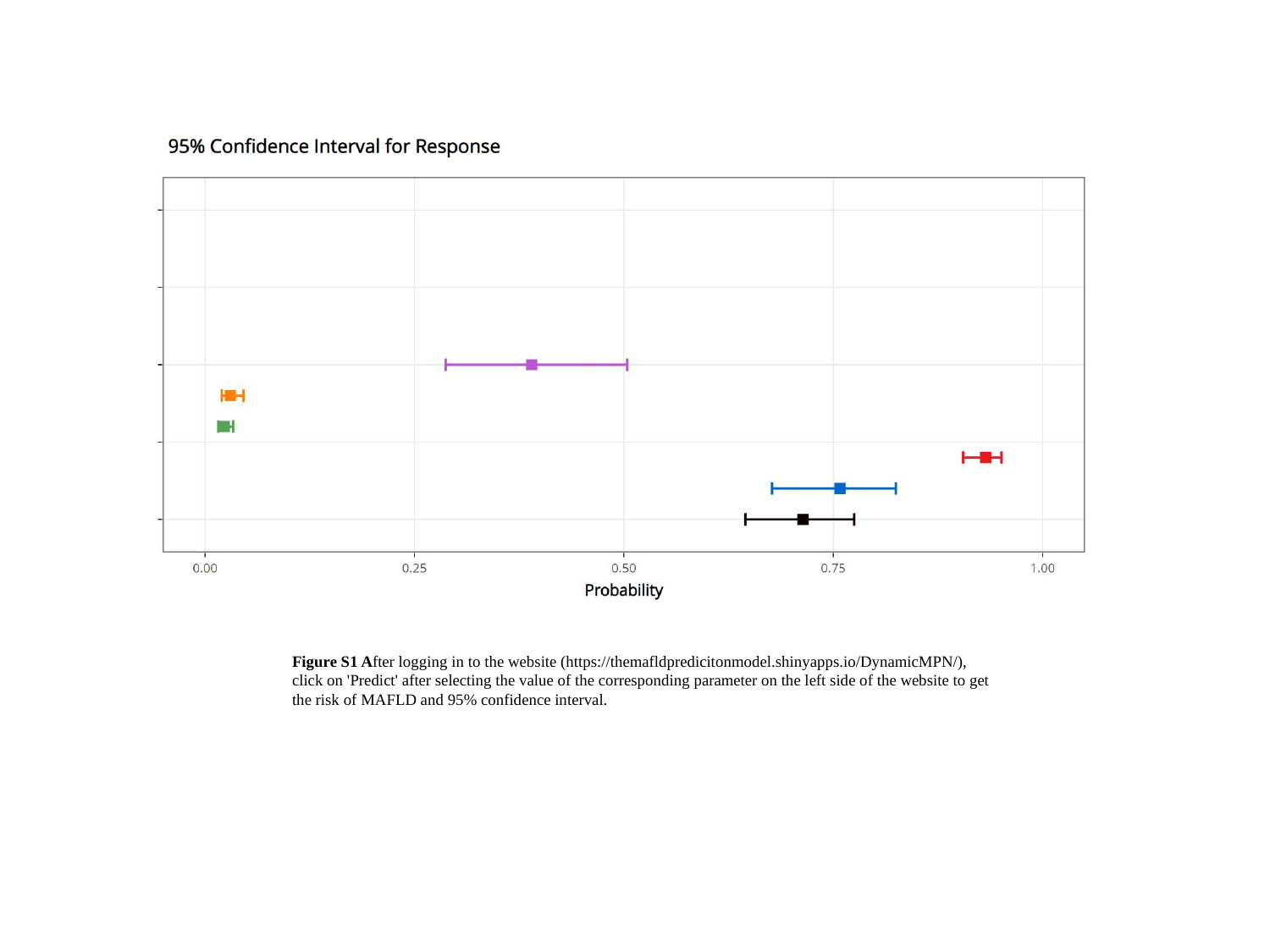

Figure S1 After logging in to the website (https://themafldpredicitonmodel.shinyapps.io/DynamicMPN/), click on 'Predict' after selecting the value of the corresponding parameter on the left side of the website to get the risk of MAFLD and 95% confidence interval.

## Slide 2
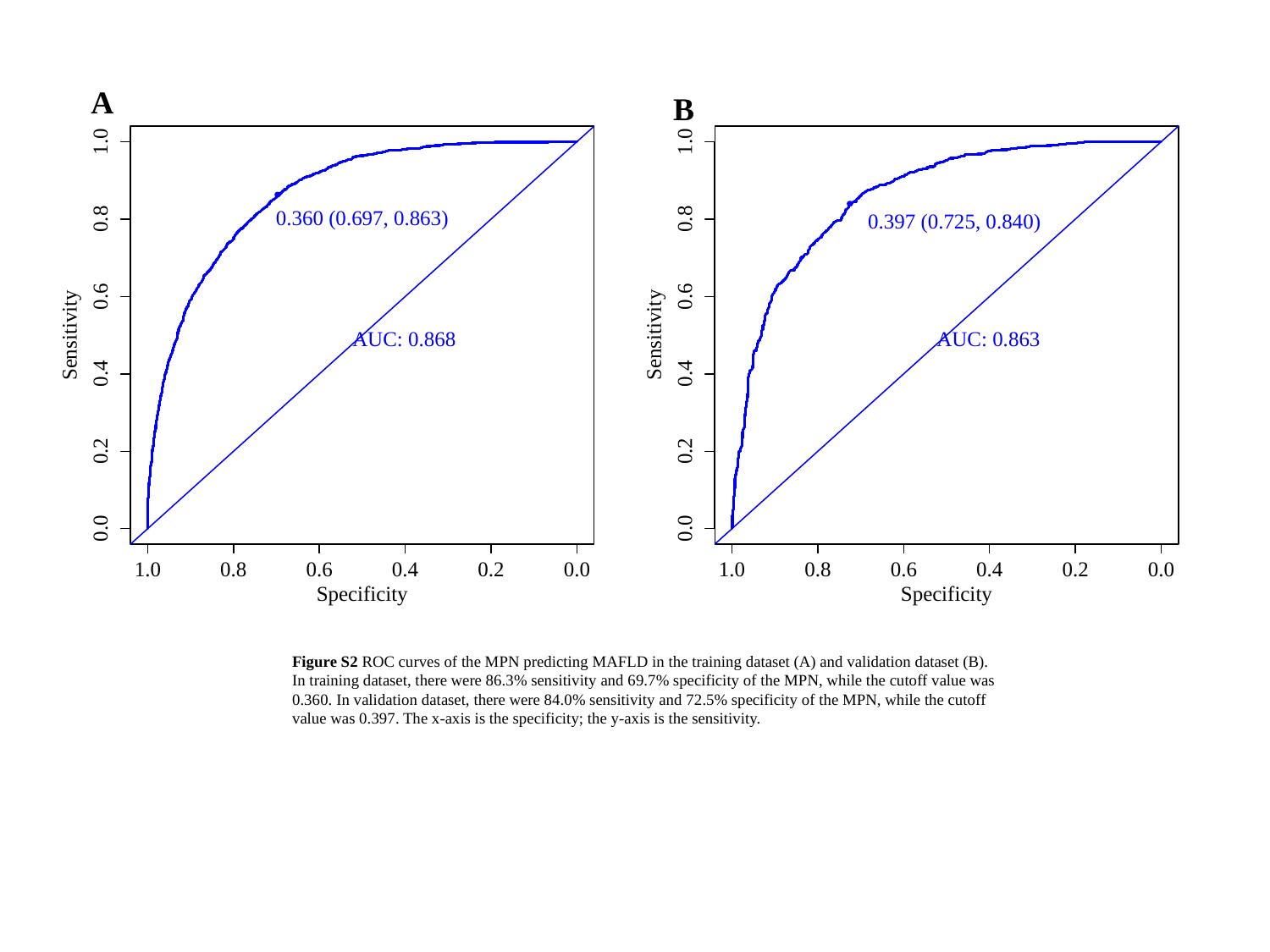

A
B
1.0
0.360 (0.697, 0.863)
0.8
0.6
Sensitivity
AUC: 0.868
0.4
0.2
0.0
1.0
0.8
0.6
0.4
0.2
0.0
Specificity
1.0
0.8
0.397 (0.725, 0.840)
0.6
Sensitivity
AUC: 0.863
0.4
0.2
0.0
1.0
0.8
0.6
0.4
0.2
0.0
Specificity
Figure S2 ROC curves of the MPN predicting MAFLD in the training dataset (A) and validation dataset (B). In training dataset, there were 86.3% sensitivity and 69.7% specificity of the MPN, while the cutoff value was 0.360. In validation dataset, there were 84.0% sensitivity and 72.5% specificity of the MPN, while the cutoff value was 0.397. The x-axis is the specificity; the y-axis is the sensitivity.

## Slide 3
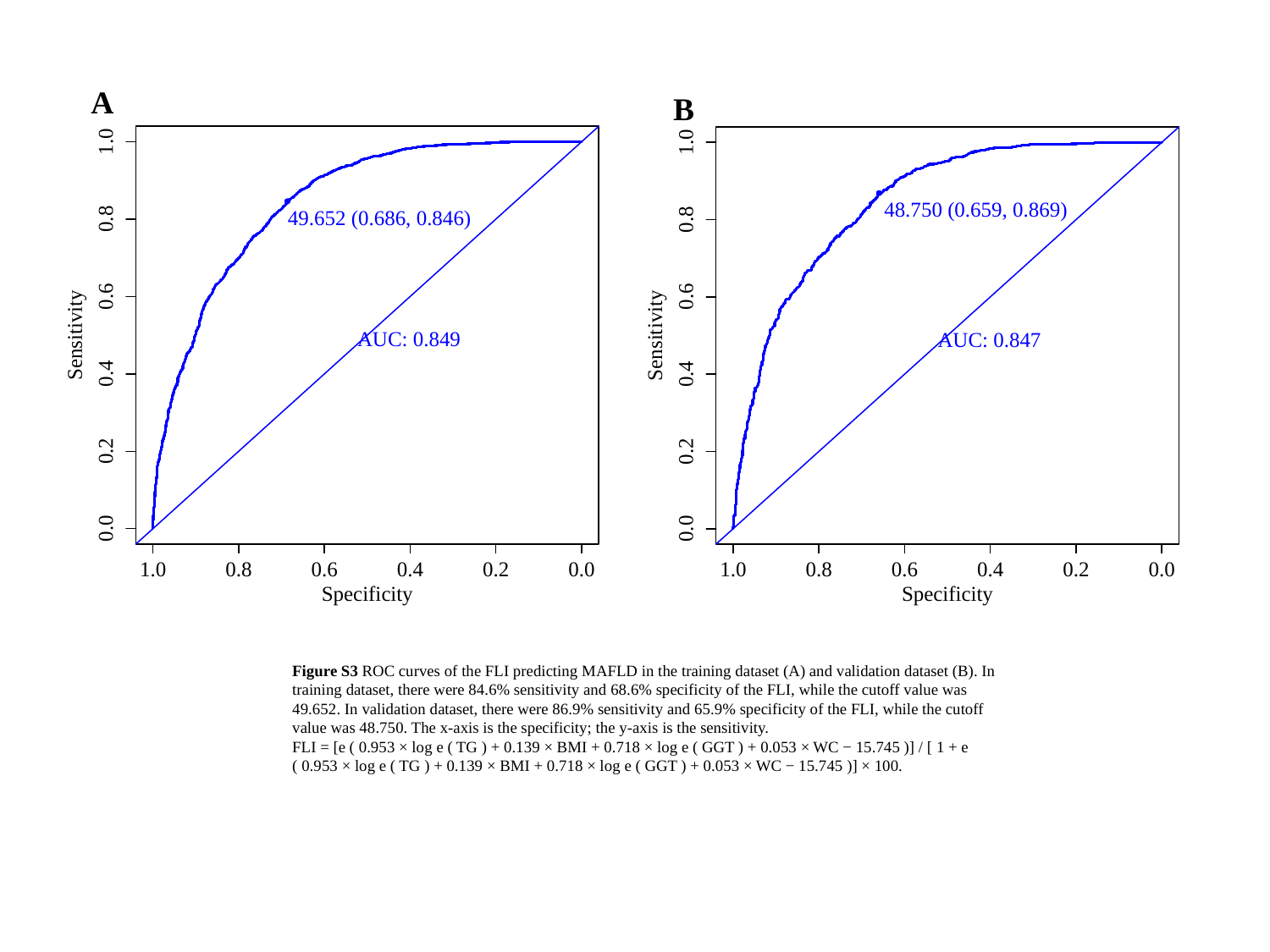

A
B
1.0
49.652 (0.686, 0.846)
0.8
0.6
Sensitivity
AUC: 0.849
0.4
0.2
0.0
1.0
0.8
0.6
0.4
0.2
0.0
Specificity
1.0
48.750 (0.659, 0.869)
0.8
0.6
Sensitivity
AUC: 0.847
0.4
0.2
0.0
1.0
0.8
0.6
0.4
0.2
0.0
Specificity
Figure S3 ROC curves of the FLI predicting MAFLD in the training dataset (A) and validation dataset (B). In training dataset, there were 84.6% sensitivity and 68.6% specificity of the FLI, while the cutoff value was 49.652. In validation dataset, there were 86.9% sensitivity and 65.9% specificity of the FLI, while the cutoff value was 48.750. The x-axis is the specificity; the y-axis is the sensitivity.
FLI = [e ( 0.953 × log e ( TG ) + 0.139 × BMI + 0.718 × log e ( GGT ) + 0.053 × WC − 15.745 )] / [ 1 + e ( 0.953 × log e ( TG ) + 0.139 × BMI + 0.718 × log e ( GGT ) + 0.053 × WC − 15.745 )] × 100.

## Slide 4
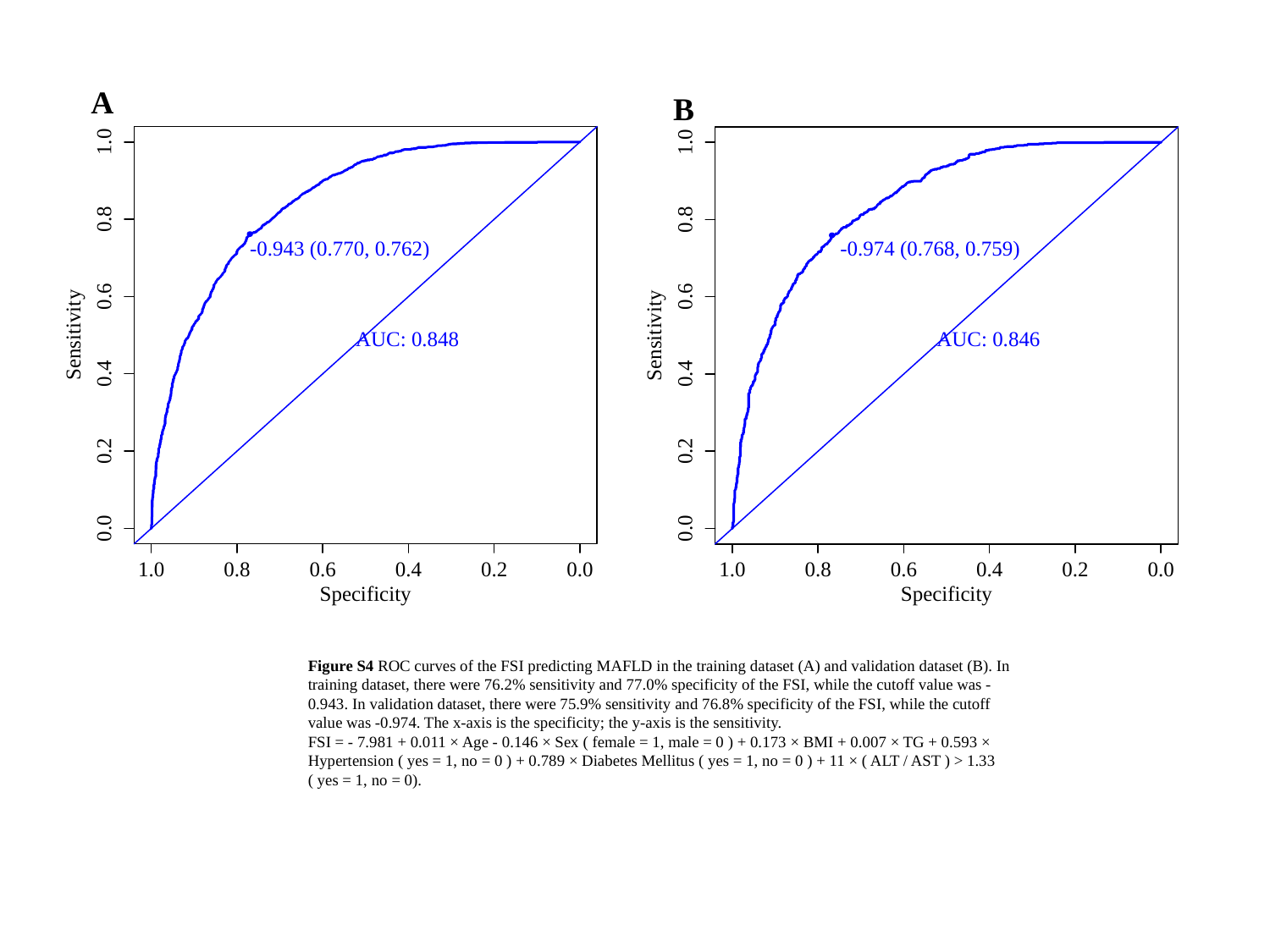

A
B
1.0
0.8
-0.943 (0.770, 0.762)
0.6
Sensitivity
AUC: 0.848
0.4
0.2
0.0
1.0
0.8
0.6
0.4
0.2
0.0
Specificity
1.0
0.8
-0.974 (0.768, 0.759)
0.6
Sensitivity
AUC: 0.846
0.4
0.2
0.0
1.0
0.8
0.6
0.4
0.2
0.0
Specificity
Figure S4 ROC curves of the FSI predicting MAFLD in the training dataset (A) and validation dataset (B). In training dataset, there were 76.2% sensitivity and 77.0% specificity of the FSI, while the cutoff value was -0.943. In validation dataset, there were 75.9% sensitivity and 76.8% specificity of the FSI, while the cutoff value was -0.974. The x-axis is the specificity; the y-axis is the sensitivity.
FSI = - 7.981 + 0.011 × Age - 0.146 × Sex ( female = 1, male = 0 ) + 0.173 × BMI + 0.007 × TG + 0.593 × Hypertension ( yes = 1, no = 0 ) + 0.789 × Diabetes Mellitus ( yes = 1, no = 0 ) + 11 × ( ALT / AST ) > 1.33 ( yes = 1, no = 0).

## Slide 5
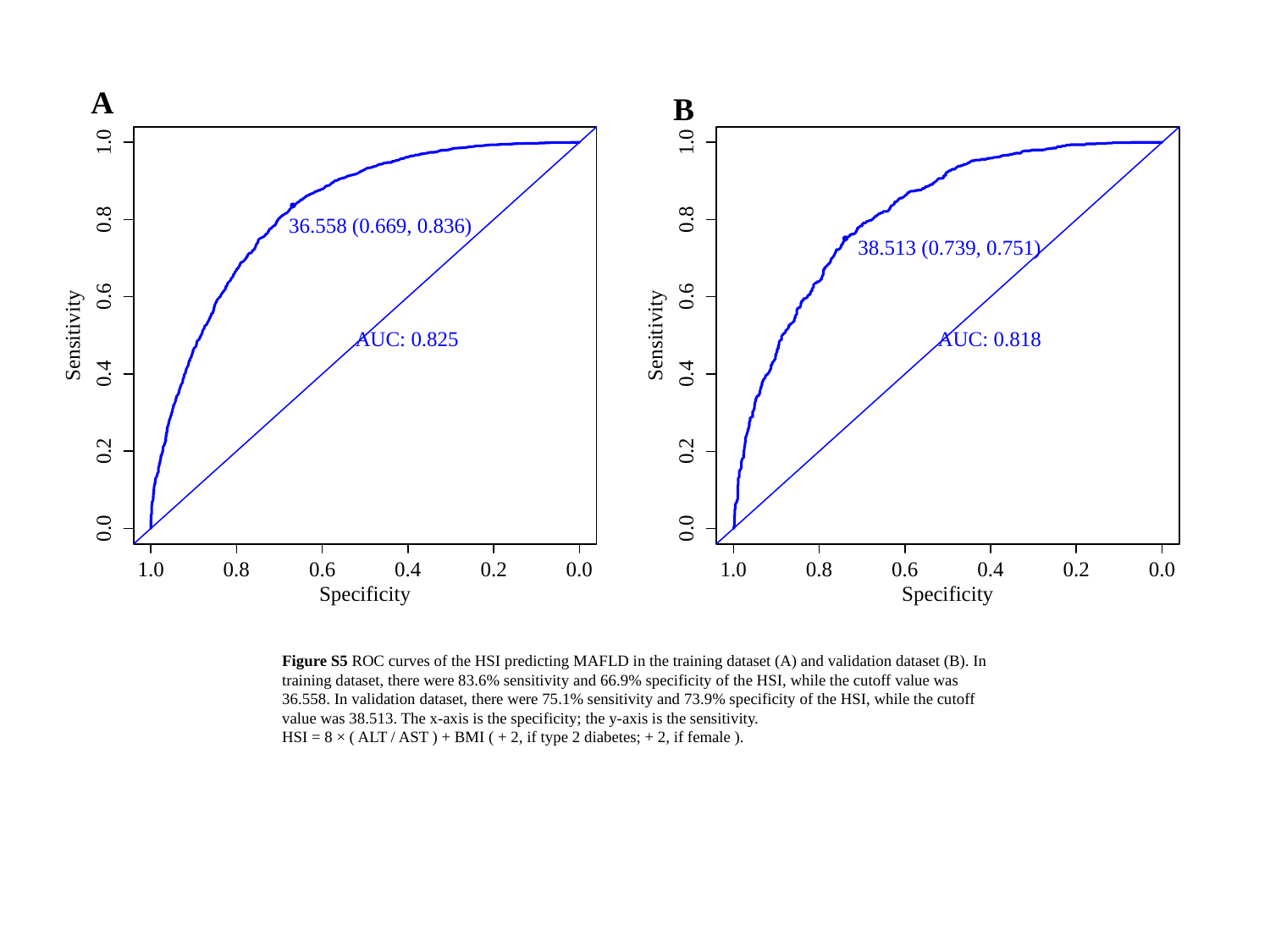

A
B
1.0
0.8
36.558 (0.669, 0.836)
0.6
Sensitivity
AUC: 0.825
0.4
0.2
0.0
1.0
0.8
0.6
0.4
0.2
0.0
Specificity
1.0
0.8
38.513 (0.739, 0.751)
0.6
Sensitivity
AUC: 0.818
0.4
0.2
0.0
1.0
0.8
0.6
0.4
0.2
0.0
Specificity
Figure S5 ROC curves of the HSI predicting MAFLD in the training dataset (A) and validation dataset (B). In training dataset, there were 83.6% sensitivity and 66.9% specificity of the HSI, while the cutoff value was 36.558. In validation dataset, there were 75.1% sensitivity and 73.9% specificity of the HSI, while the cutoff value was 38.513. The x-axis is the specificity; the y-axis is the sensitivity.
HSI = 8 × ( ALT / AST ) + BMI ( + 2, if type 2 diabetes; + 2, if female ).

## Slide 6
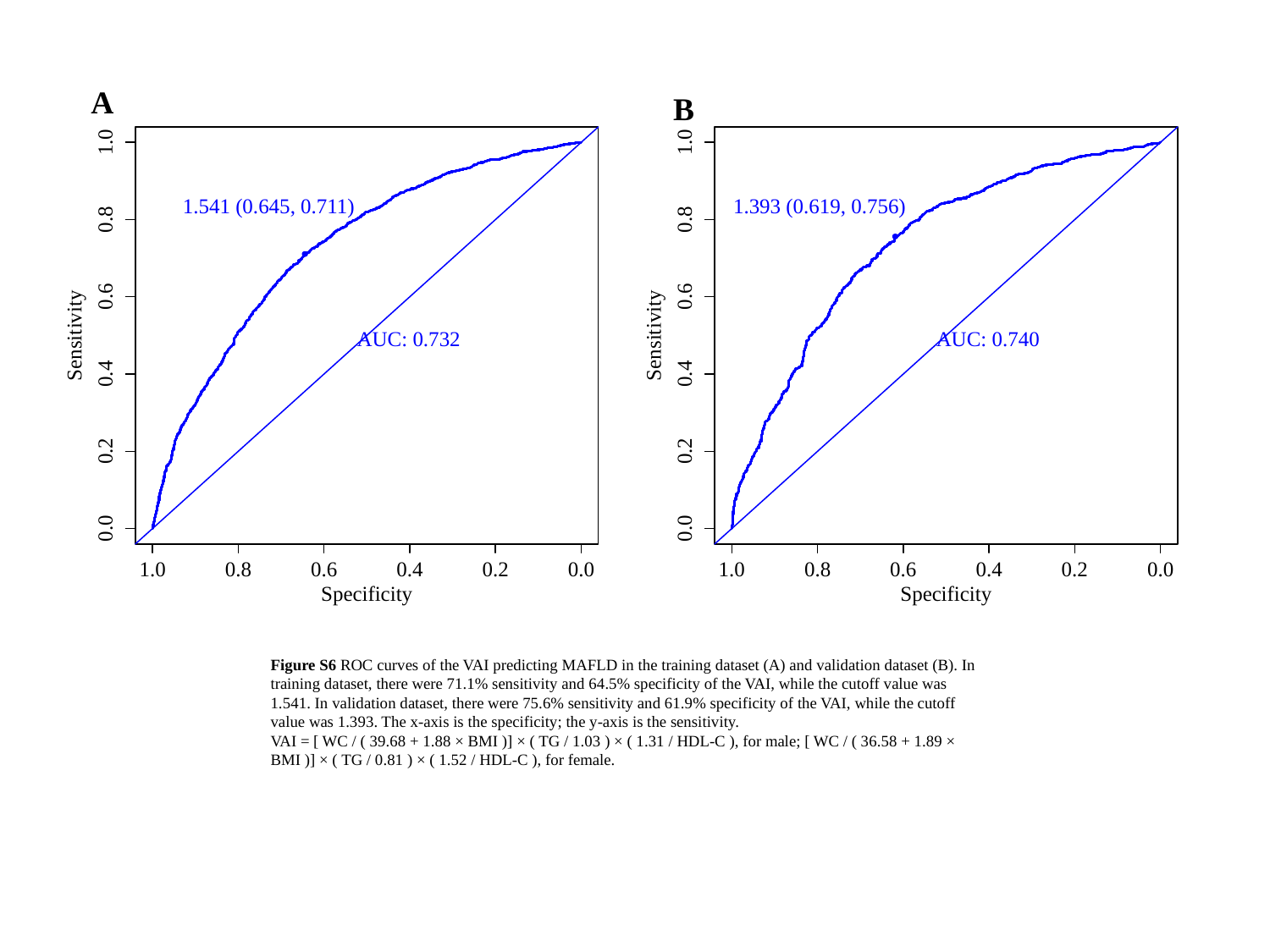

A
B
1.0
1.541 (0.645, 0.711)
0.8
0.6
Sensitivity
AUC: 0.732
0.4
0.2
0.0
1.0
0.8
0.6
0.4
0.2
0.0
Specificity
1.0
1.393 (0.619, 0.756)
0.8
0.6
Sensitivity
AUC: 0.740
0.4
0.2
0.0
1.0
0.8
0.6
0.4
0.2
0.0
Specificity
Figure S6 ROC curves of the VAI predicting MAFLD in the training dataset (A) and validation dataset (B). In training dataset, there were 71.1% sensitivity and 64.5% specificity of the VAI, while the cutoff value was 1.541. In validation dataset, there were 75.6% sensitivity and 61.9% specificity of the VAI, while the cutoff value was 1.393. The x-axis is the specificity; the y-axis is the sensitivity.
VAI = [ WC / ( 39.68 + 1.88 × BMI )] × ( TG / 1.03 ) × ( 1.31 / HDL-C ), for male; [ WC / ( 36.58 + 1.89 × BMI )] × ( TG / 0.81 ) × ( 1.52 / HDL-C ), for female.

## Slide 7
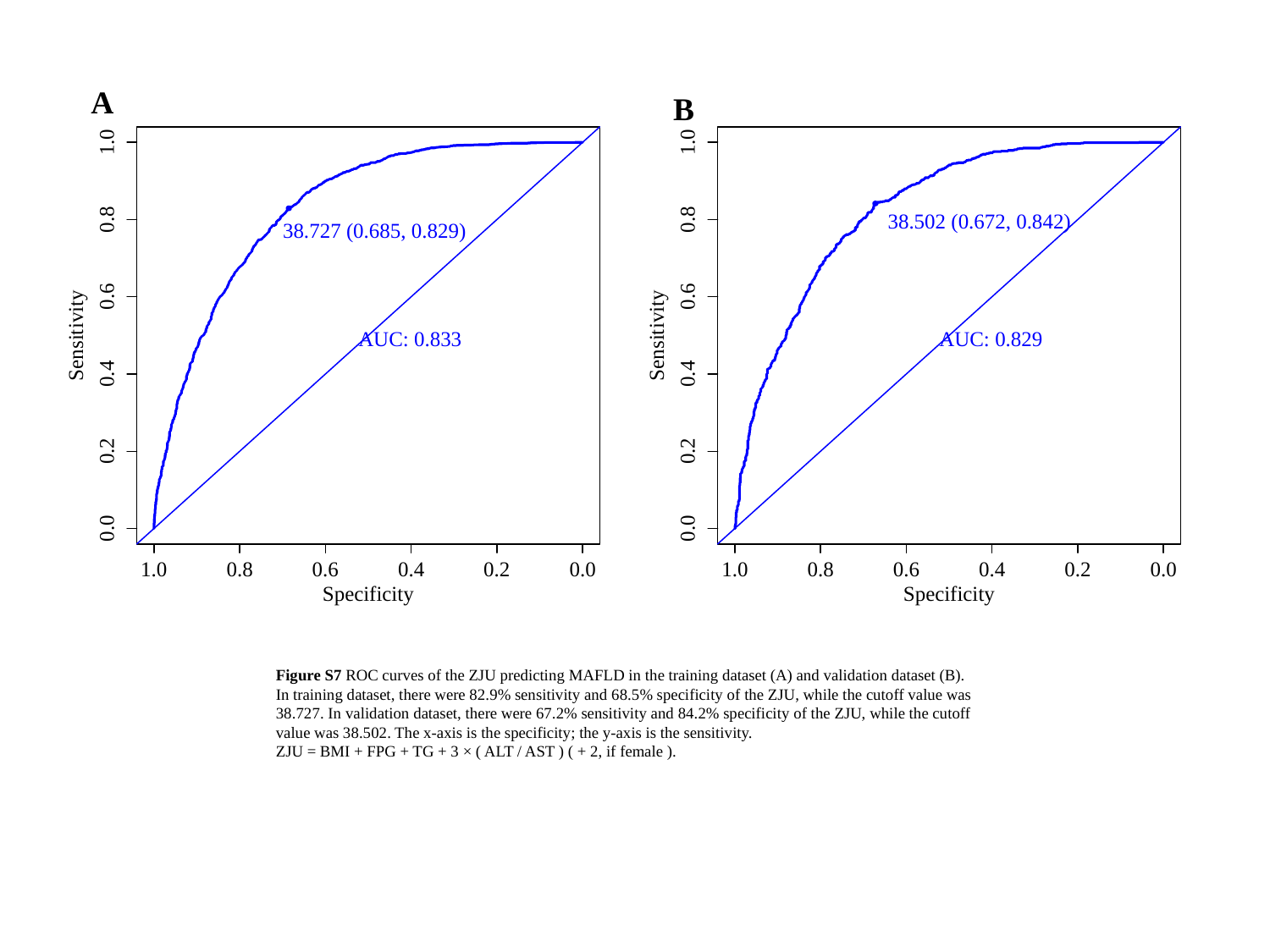

A
B
1.0
0.8
38.727 (0.685, 0.829)
0.6
Sensitivity
AUC: 0.833
0.4
0.2
0.0
1.0
0.8
0.6
0.4
0.2
0.0
Specificity
1.0
0.8
38.502 (0.672, 0.842)
0.6
Sensitivity
AUC: 0.829
0.4
0.2
0.0
1.0
0.8
0.6
0.4
0.2
0.0
Specificity
Figure S7 ROC curves of the ZJU predicting MAFLD in the training dataset (A) and validation dataset (B). In training dataset, there were 82.9% sensitivity and 68.5% specificity of the ZJU, while the cutoff value was 38.727. In validation dataset, there were 67.2% sensitivity and 84.2% specificity of the ZJU, while the cutoff value was 38.502. The x-axis is the specificity; the y-axis is the sensitivity.
ZJU = BMI + FPG + TG + 3 × ( ALT / AST ) ( + 2, if female ).

## Slide 8
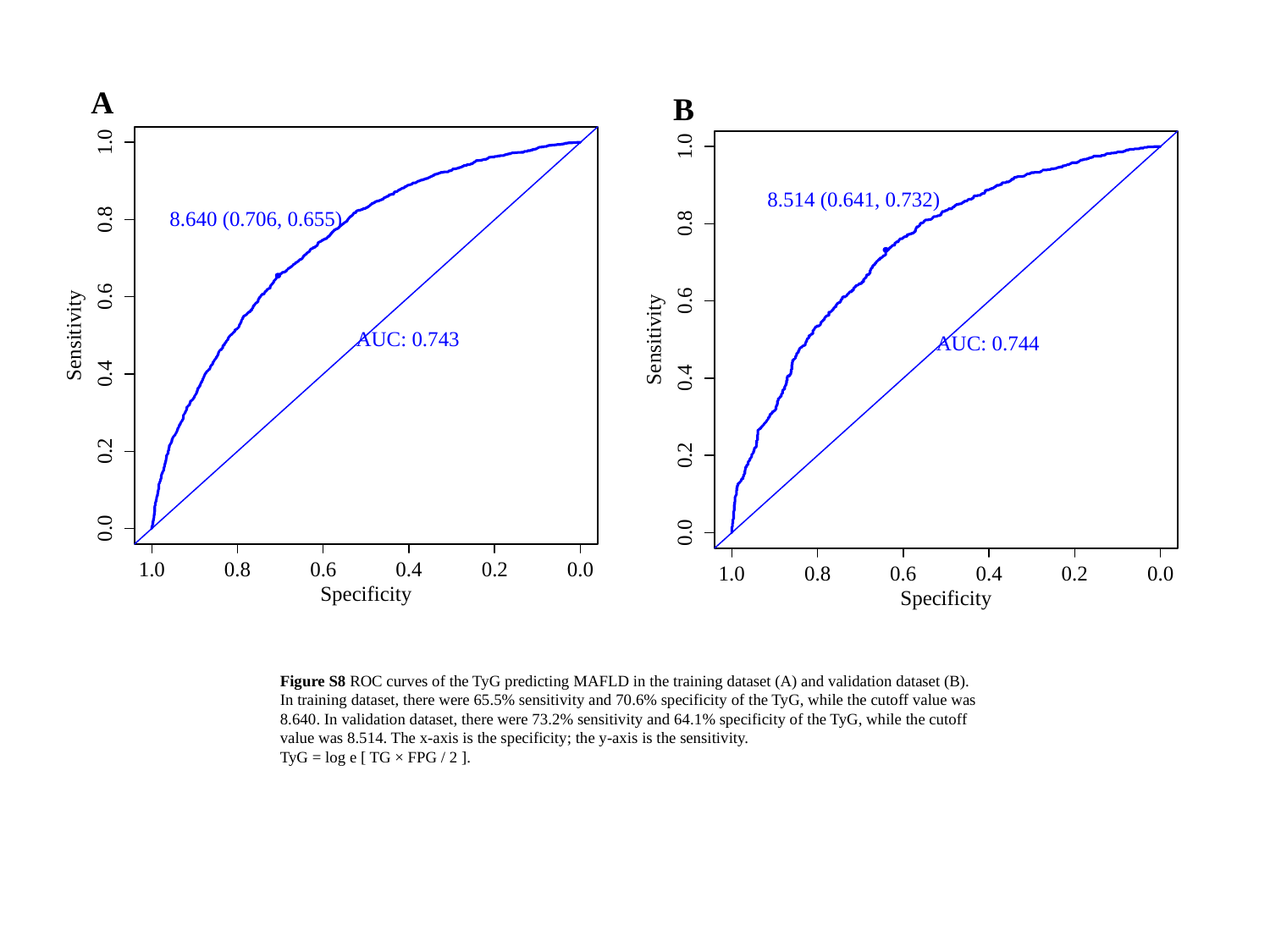

A
B
1.0
8.640 (0.706, 0.655)
0.8
0.6
Sensitivity
AUC: 0.743
0.4
0.2
0.0
1.0
0.8
0.6
0.4
0.2
0.0
Specificity
1.0
8.514 (0.641, 0.732)
0.8
0.6
Sensitivity
AUC: 0.744
0.4
0.2
0.0
1.0
0.8
0.6
0.4
0.2
0.0
Specificity
Figure S8 ROC curves of the TyG predicting MAFLD in the training dataset (A) and validation dataset (B). In training dataset, there were 65.5% sensitivity and 70.6% specificity of the TyG, while the cutoff value was 8.640. In validation dataset, there were 73.2% sensitivity and 64.1% specificity of the TyG, while the cutoff value was 8.514. The x-axis is the specificity; the y-axis is the sensitivity.
TyG = log e [ TG × FPG / 2 ].

## Slide 9
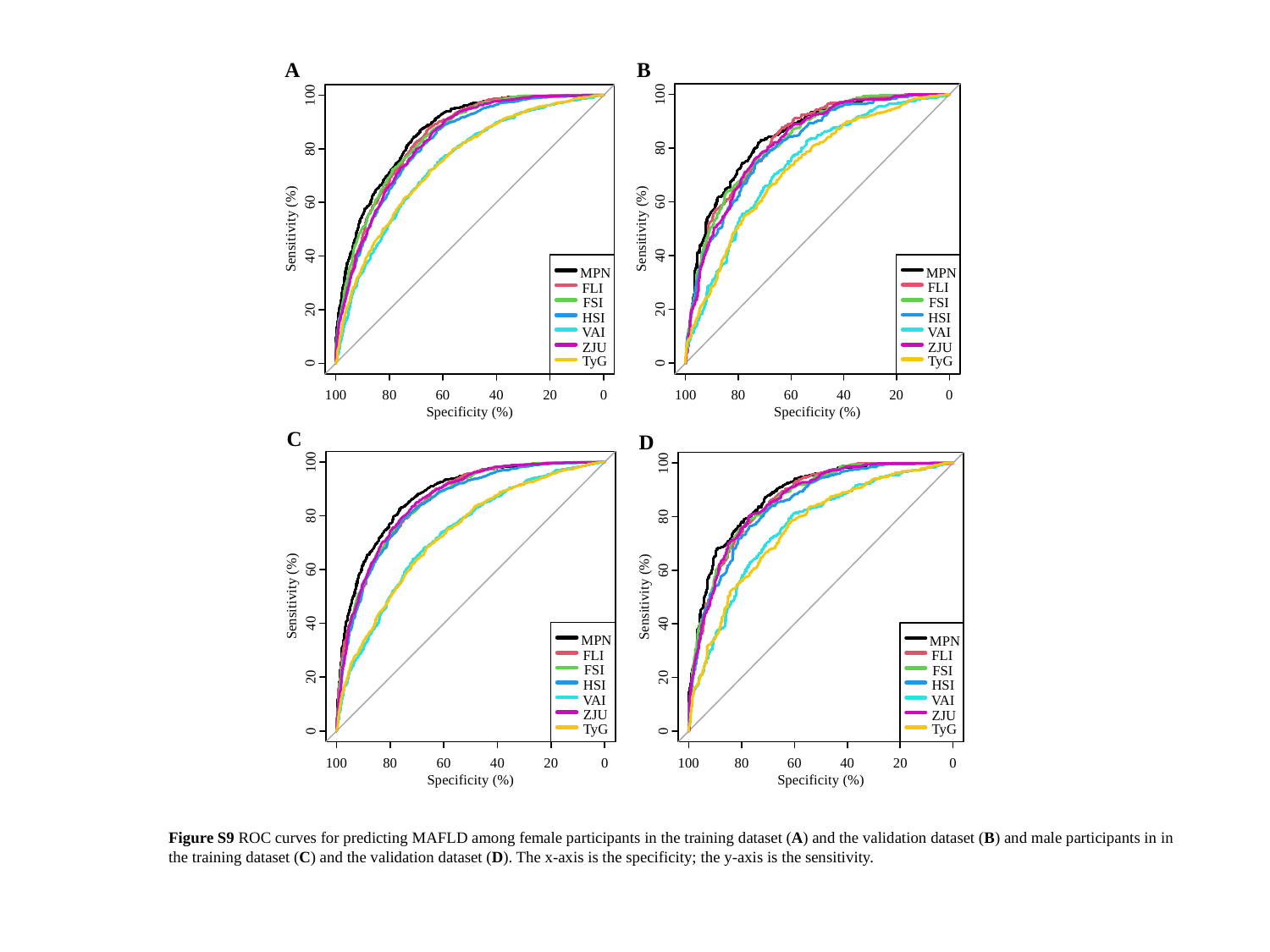

B
A
100
80
60
Sensitivity (%)
40
20
0
100
80
60
40
20
0
Specificity (%)
MPN
FLI
FSI
HSI
VAI
ZJU
TyG
100
80
60
Sensitivity (%)
40
MPN
FLI
FSI
HSI
VAI
ZJU
TyG
20
0
100
80
60
40
20
0
Specificity (%)
C
D
100
80
60
Sensitivity (%)
40
20
0
100
80
60
40
20
0
Specificity (%)
MPN
FLI
FSI
HSI
VAI
ZJU
TyG
100
80
60
Sensitivity (%)
40
20
0
100
80
60
40
20
0
Specificity (%)
MPN
FLI
FSI
HSI
VAI
ZJU
TyG
Figure S9 ROC curves for predicting MAFLD among female participants in the training dataset (A) and the validation dataset (B) and male participants in in the training dataset (C) and the validation dataset (D). The x-axis is the specificity; the y-axis is the sensitivity.

## Slide 10
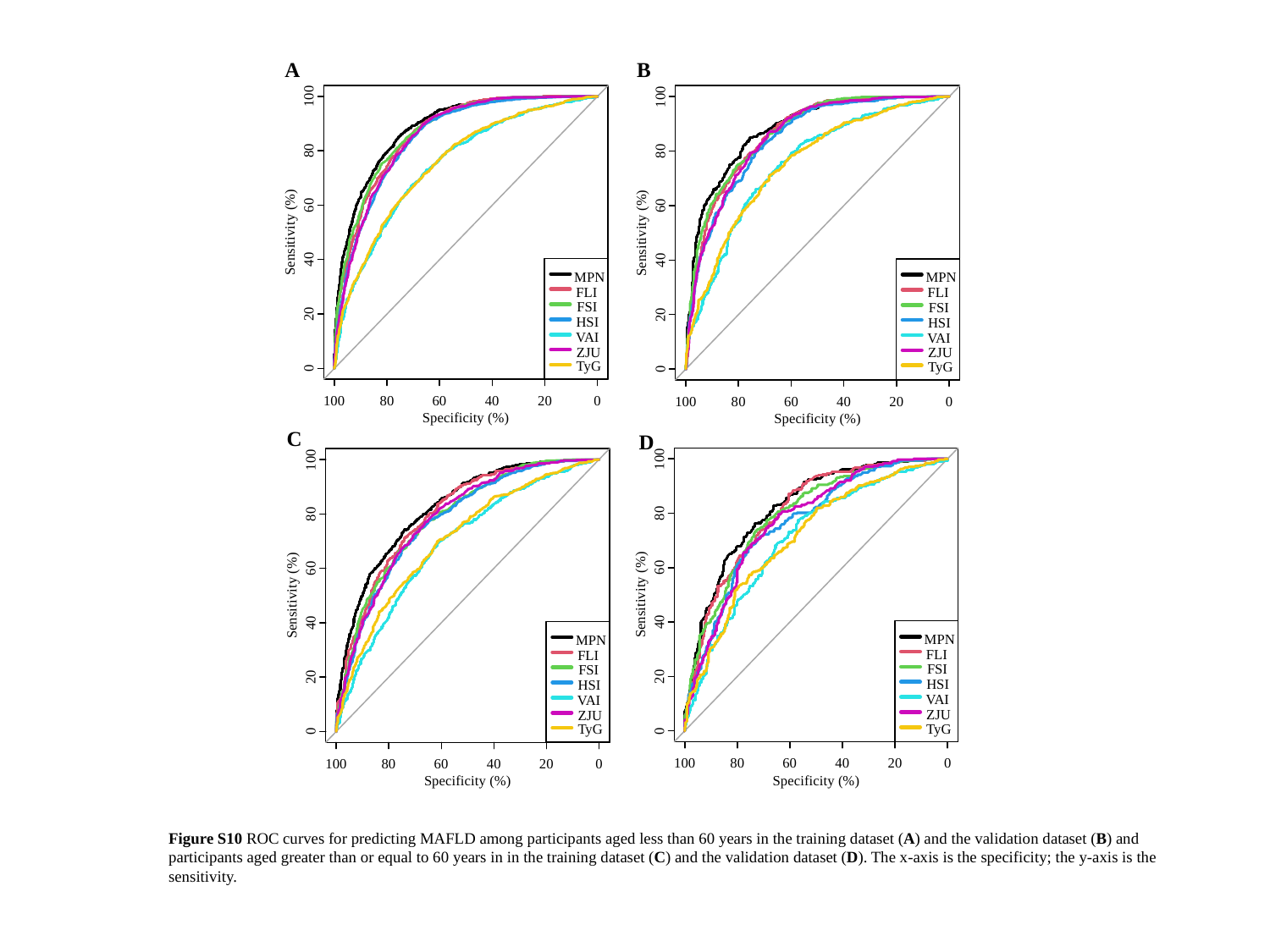

B
A
100
80
60
Sensitivity (%)
40
20
0
100
80
60
40
20
0
Specificity (%)
MPN
FLI
FSI
HSI
VAI
ZJU
TyG
100
80
60
Sensitivity (%)
40
20
0
100
80
60
40
20
0
Specificity (%)
MPN
FLI
FSI
HSI
VAI
ZJU
TyG
C
100
80
60
Sensitivity (%)
40
20
0
100
80
60
40
20
0
Specificity (%)
MPN
FLI
FSI
HSI
VAI
ZJU
TyG
D
100
80
60
Sensitivity (%)
40
20
0
100
80
60
40
20
0
Specificity (%)
MPN
FLI
FSI
HSI
VAI
ZJU
TyG
Figure S10 ROC curves for predicting MAFLD among participants aged less than 60 years in the training dataset (A) and the validation dataset (B) and participants aged greater than or equal to 60 years in in the training dataset (C) and the validation dataset (D). The x-axis is the specificity; the y-axis is the sensitivity.

## Slide 11
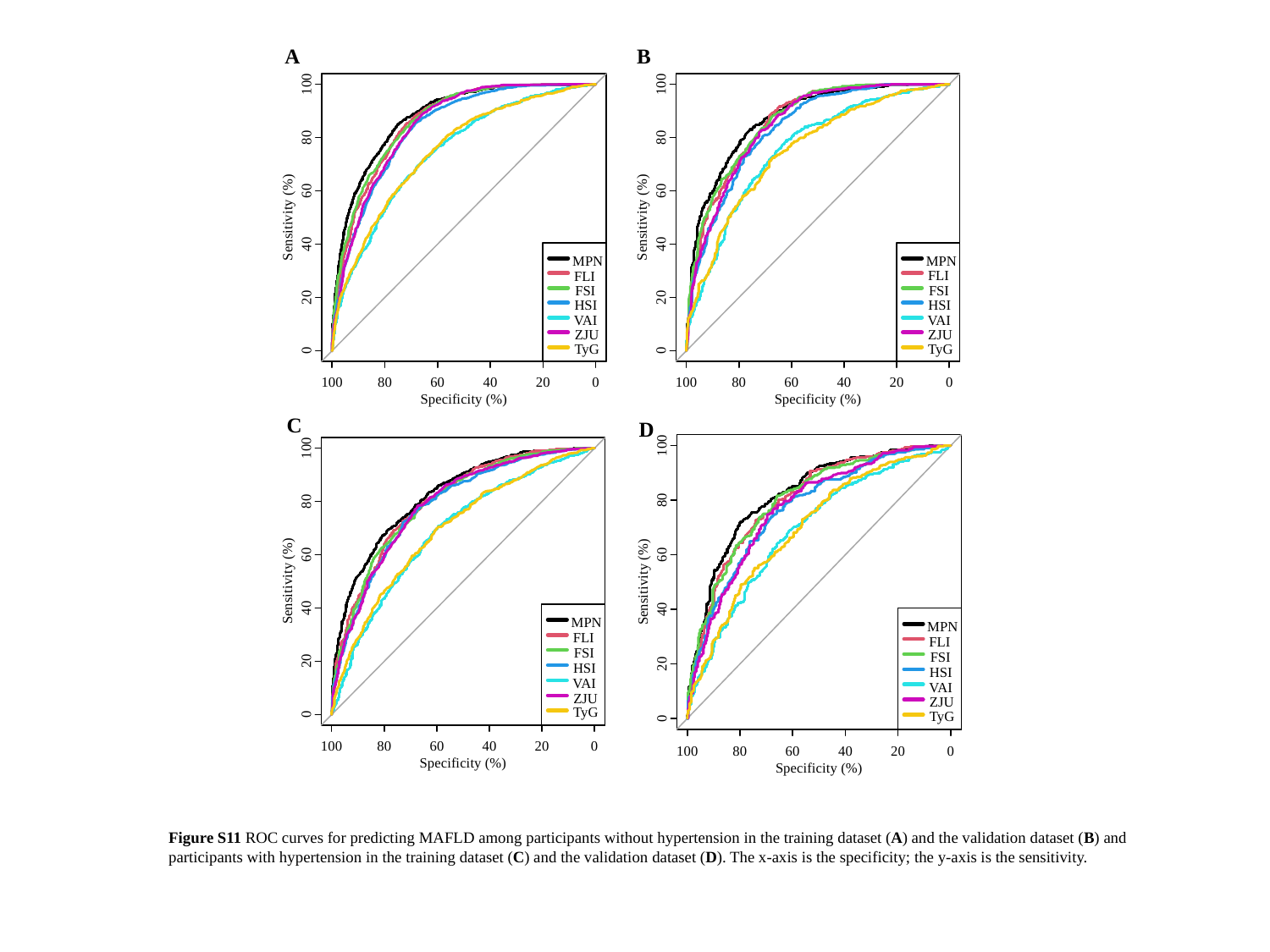

B
A
100
80
60
Sensitivity (%)
40
20
0
100
80
60
40
20
0
Specificity (%)
MPN
FLI
FSI
HSI
VAI
ZJU
TyG
100
80
60
Sensitivity (%)
40
20
0
100
80
60
40
20
0
Specificity (%)
MPN
FLI
FSI
HSI
VAI
ZJU
TyG
C
100
80
60
Sensitivity (%)
40
20
0
100
80
60
40
20
0
Specificity (%)
D
100
80
60
Sensitivity (%)
40
20
0
100
80
60
40
20
0
Specificity (%)
MPN
FLI
FSI
HSI
VAI
ZJU
TyG
MPN
FLI
FSI
HSI
VAI
ZJU
TyG
Figure S11 ROC curves for predicting MAFLD among participants without hypertension in the training dataset (A) and the validation dataset (B) and participants with hypertension in the training dataset (C) and the validation dataset (D). The x-axis is the specificity; the y-axis is the sensitivity.

## Slide 12
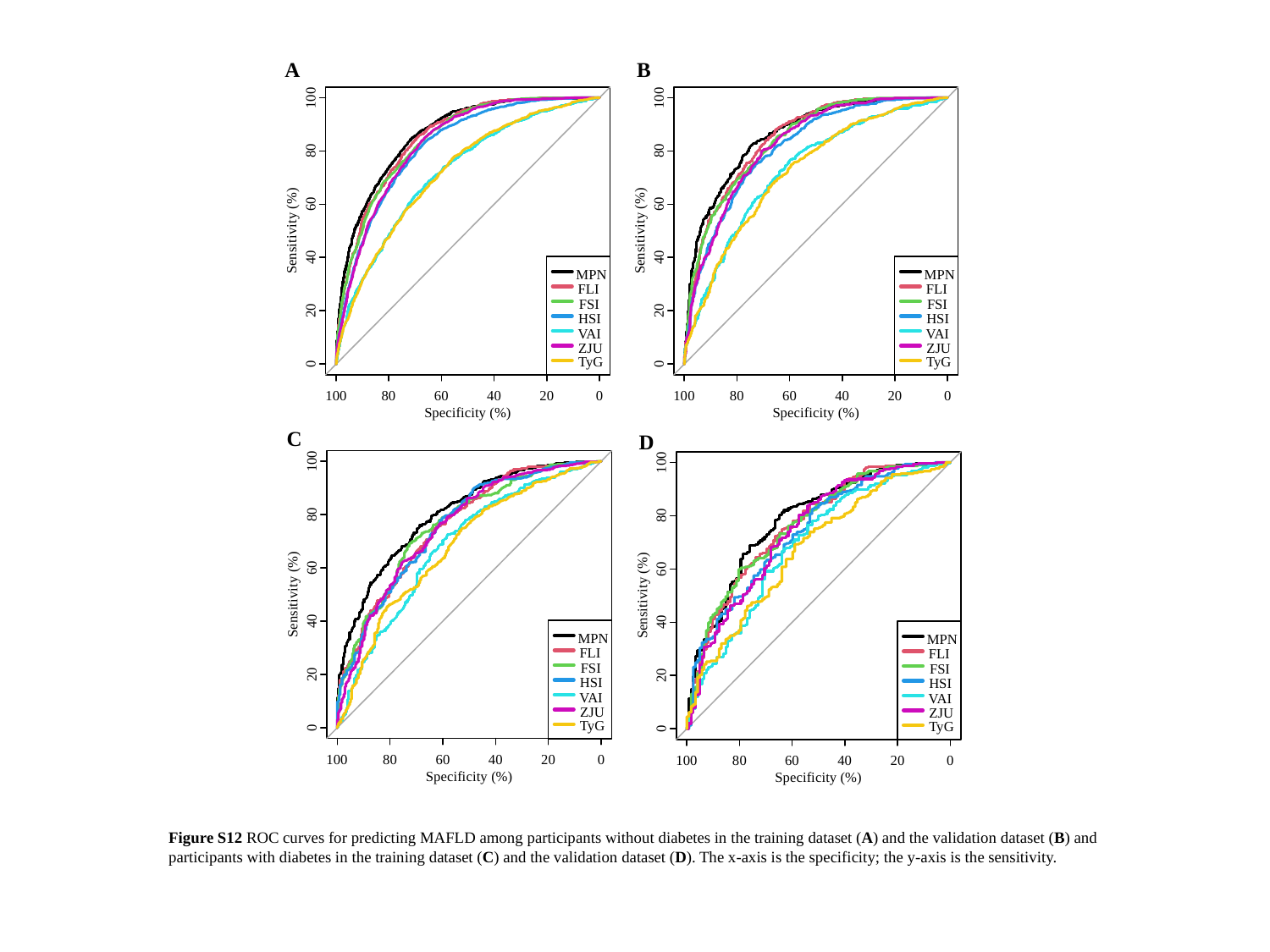

B
A
100
80
60
Sensitivity (%)
40
20
0
100
80
60
40
20
0
Specificity (%)
100
80
60
Sensitivity (%)
40
20
0
100
80
60
40
20
0
Specificity (%)
MPN
FLI
FSI
HSI
VAI
ZJU
TyG
MPN
FLI
FSI
HSI
VAI
ZJU
TyG
C
D
100
80
60
Sensitivity (%)
40
20
0
100
80
60
40
20
0
Specificity (%)
100
80
60
Sensitivity (%)
40
20
0
100
80
60
40
20
0
Specificity (%)
MPN
FLI
FSI
HSI
VAI
ZJU
TyG
MPN
FLI
FSI
HSI
VAI
ZJU
TyG
Figure S12 ROC curves for predicting MAFLD among participants without diabetes in the training dataset (A) and the validation dataset (B) and participants with diabetes in the training dataset (C) and the validation dataset (D). The x-axis is the specificity; the y-axis is the sensitivity.
