# Supplementary material for: Development and validation of a new nomogram to screen for MAFLD
Source: Lipids Health Dis. 2022 Dec 8;21:133. doi: 10.1186/s12944-022-01748-1 (PMC9730620; doi:10.1186/s12944-022-01748-1)
Supplement: Supplementary file 2 — Additional file 2. Supplementary tables [file 12944_2022_1748_MOESM2_ESM.docx]

**Table S1** Baseline characteristics of participants in the training set and validation set

|  | Training set (n = 5112) | Validation set (n = 2188) | *P* value |
| --- | --- | --- | --- |
| Age (years) | 48.81 ± 18.07 | 49.10 ± 18.04 | 0.527 |
| Sex, n (%) |  |  | 0.682 |
| Female | 2572 (50.31) | 1113 (50.87) |  |
| Male | 2540 (49.69) | 1075 (49.13) |  |
| Race, n (%) |  |  | 0.395 |
| Non-Hispanic Asian | 621 (12.15) | 263 (12.02) |  |
| Non-Hispanic Black | 1249 (24.43) | 568 (25.96) |  |
| Non-Hispanic White | 1813 (35.47) | 750 (34.28) |  |
| Other Hispanic | 531 (10.39) | 232 (10.60) |  |
| Mexican American | 660 (12.91) | 258 (11.79) |  |
| Other | 238 ± 4.66 | 117 ± 5.35 |  |
| AC (cm) | 33.50 ± 5.26 | 33.63 ± 5.18 | 0.348 |
| HC (cm) | 106.81 ± 14.19 | 106.99 ± 14.07 | 0.622 |
| WC (cm) | 99.98 ± 17.08 | 100.27 ± 16.66 | 0.505 |
| BMI (kg/m^2^) | 29.60 ± 7.08 | 29.71 ± 6.98 | 0.555 |
| WBC (×10^9^/l) | 7.26 ± 5.99 | 7.09 ± 2.07 | 0.2 |
| LYM (×10^9^/l) | 2.28 ± 5.15 | 2.18 ± 0.71 | 0.363 |
| NEU (×10^9^/l) | 4.16 ± 1.71 | 4.11 ± 1.63 | 0.179 |
| HB (g/l) | 14.05 ± 1.54 | 14.00 ± 1.55 | 0.239 |
| PLT (×10^9^/l) | 246.58 ± 64.85 | 246.23 ± 64.98 | 0.831 |
| FPG (mmol/l) | 5.60 ± 1.94 | 5.66 ± 1.98 | 0.212 |
| TG (mmol/l) | 1.55 ± 1.13 | 1.54 ± 1.12 | 0.857 |
| TyG | 8.64 ± 0.65 | 8.64 ± 0.66 | 0.851 |
| ALT (u/l) | 22.25 ± 17.35 | 22.50 ± 22.37 | 0.612 |
| AST (u/l) | 21.86 ± 12.74 | 21.95 ± 17.74 | 0.802 |
| ALT/AST ratio | 0.99 ± 0.35 | 1.00 ± 0.36 | 0.174 |
| TC (mmol/l) | 4.79 ± 1.04 | 4.79 ± 1.06 | 0.976 |
| HDL-C (mmol/l) | 1.38 ± 0.41 | 1.38 ± 0.40 | 0.905 |
| GGT (u/l) | 31.61 ± 54.89 | 31.59 ± 43.26 | 0.989 |
| ALP (u/l) | 78.03 ± 26.38 | 76.95 ± 24.36 | 0.1 |
| ALB (g/l) | 40.82 ± 3.33 | 40.73 ± 3.37 | 0.303 |
| GLB (g/l) | 30.89 ± 4.27 | 30.86 ± 4.42 | 0.804 |
| TP (g/l) | 71.71 ± 4.43 | 71.59 ± 4.42 | 0.308 |
| TBIL (mg/dl) | 7.84 ± 4.73 | 7.89 ± 4.77 | 0.667 |
| hsCRP (mg/l) | 3.91 ± 8.65 | 3.95 ± 7.40 | 0.843 |
| CRE (mg/dl) | 78.85 ± 38.62 | 79.47 ± 40.56 | 0.536 |
| BUN (mg/dl) | 5.24 ± 2.00 | 5.27 ± 2.06 | 0.533 |
| eGFR (mg/min/1.73m^2^) | 95.79 ± 23.58 | 95.13 ± 23.61 | 0.268 |
| Hypertension, n (%) |  |  | 0.717 |
| No | 3272 (64.01) | 1390 (63.53) |  |
| Yes | 1840 (35.99) | 798 (36.47) |  |
| Diabetes, n (%) |  |  | 0.679 |
| No | 4135 (80.89) | 1760 (80.44) |  |
| Yes | 977 (19.11) | 428 (19.56) |  |
| Smoking, n (%) |  |  | 0.378 |
| Never | 3039 (59.45) | 1328 (60.69) |  |
| Former | 1157 (22.63) | 497 (22.71) |  |
| Current | 916 (17.92) | 363 (16.59) |  |
| MAFLD, n (%) |  |  | 0.731 |
| No | 2788 (54.54) | 1183 (54.07) |  |
| Yes | 2324 (45.46) | 1005 (45.93) |  |
| FLI | 55.04 ± 32.78 | 55.73 ± 32.36 | 0.404 |
| HSI | 38.91 ± 8.53 | 39.13 ± 8.49 | 0.301 |
| VAI | 2.17 ± 2.22 | 2.18 ± 2.34 | 0.839 |
| FSI | -0.90 ± 1.88 | -0.87 ± 1.86 | 0.523 |
| ZJU | 40.72 ± 8.34 | 40.93 ± 8.34 | 0.321 |

Data are presented as means ± SD and absolute or relative percentages.

**Table S2** Logistic regression analysis of the predictors for MALFD

| Intercept and variables | Estimate | Std. Error | *Z* value | *P* value | OR | CI (2.5%) | CI (97.5%) |
| --- | --- | --- | --- | --- | --- | --- | --- |
| Intercept | -18.004 | 0.703 | -25.603 | <0.001 | 0.000 | 0.000 | 0.000 |
| Age | 0.014 | 0.003 | 5.239 | <0.001 | 1.014 | 1.009 | 1.020 |
| Race |  |  |  |  |  |  |  |
| Non-Hispanic Black | Ref | | | | | | |
| Non-Hispanic White | 0.415 | 0.105 | 3.974 | <0.001 | 1.515 | 1.235 | 1.860 |
| Other Hispanic | 0.404 | 0.139 | 2.900 | 0.004 | 1.498 | 1.140 | 1.969 |
| Non-Hispanic Asian | 0.737 | 0.139 | 5.306 | <0.001 | 2.089 | 1.592 | 2.744 |
| Mexican American | 1.036 | 0.133 | 7.758 | <0.001 | 2.817 | 2.171 | 3.664 |
| Other | 0.183 | 0.194 | 0.946 | 0.344 | 1.201 | 0.820 | 1.756 |
| AC | 0.034 | 0.016 | 2.175 | 0.030 | 1.035 | 1.003 | 1.068 |
| WC | 0.050 | 0.006 | 8.115 | <0.001 | 1.052 | 1.039 | 1.064 |
| BMI | 0.056 | 0.016 | 3.456 | <0.001 | 1.057 | 1.025 | 1.091 |
| ALT/AST ratio | 1.322 | 0.122 | 10.834 | <0.001 | 3.751 | 2.958 | 4.773 |
| TyG | 0.850 | 0.071 | 11.948 | <0.001 | 2.341 | 2.038 | 2.694 |
| Hypertension | 0.204 | 0.084 | 2.424 | 0.015 | 1.227 | 1.040 | 1.447 |
| Diabetes | 0.276 | 0.103 | 2.665 | 0.008 | 1.317 | 1.076 | 1.614 |

**Table S3** Performance assessment of the MPN and other existing models for the prediction of MAFLD among male participants

| Models | AUC (95% CI) | *P**  values | SEN (95% CI) | SPE (95% CI) | PPV (95% CI) | NPV (95% CI) | Cutoff values |
| --- | --- | --- | --- | --- | --- | --- | --- |
| **Male in the training set** |  |  |  |  |  |  |  |
| MPN | 0.875 (0.861-0.888) | - | 0.826 (0.805-0.847) | 0.765 (0.742-0.788) | 0.781 (0.759-0.803) | 0.812 (0.790-0.834) | 0.478 |
| FLI | 0.855 (0.841-0.869) | <0.001 | 0.787 (0.764-0.809) | 0.750 (0.726-0.774) | 0.762 (0.739-0.785) | 0.776 (0.752-0.799) | 61.938 |
| HSI | 0.844 (0.829-0.853) | <0.001 | 0.787 (0.765-0.810) | 0.755 (0.731-0.779) | 0.765 (0.742-0.788) | 0.778 (0.754-0.801) | 37.117 |
| VAI | 0.730 (0.711-0.749) | <0.001 | 0.684 (0.659-0.710) | 0.671 (0.645-0.697) | 0.679 (0.654-0.705) | 0.677 (0.651-0.703) | 1.526 |
| FSI | 0.852 (0.838-0.867) | <0.001 | 0.787 (0.765-0.810) | 0.760 (0.737-0.784) | 0.769 (0.747-0.792) | 0.779 (0.756-0.802) | -0.940 |
| ZJU | 0.856 (0.842-0.871) | <0.001 | 0.835 (0.815-0.855) | 0.719 (0.694-0.744) | 0.751 (0.729-0.774) | 0.811 (0.788-0.834) | 37.795 |
| TyG | 0.730 (0.711-0.750) | <0.001 | 0.687 (0.661-0.712) | 0.659 (0.633-0.685) | 0.672 (0.646-0.697) | 0.674 (0.648-0.700) | 8.642 |
| **Male in the validation set** |  |  |  |  |  |  |  |
| MPN | 0.878 (0.858-0.898) | - | 0.871 (0.843-0.899) | 0.712 (0.673-0.750) | 0.756 (0.722-0.790) | 0.844 (0.810-0.877) | 0.401 |
| FLI | 0.862 (0.841-0.884) | 0.010 | 0.761 (0.725-0.797) | 0.795 (0.760-0.829) | 0.792 (0.757-0.826) | 0.764 (0.729-0.800) | 64.178 |
| HSI | 0.845 (0.822-0.868) | <0.001 | 0.765 (0.729-0.800) | 0.770 (0.734-0.806) | 0.773 (0.738-0.809) | 0.762 (0.726-0.798) | 37.668 |
| VAI | 0.758 (0.729-0.787) | <0.001 | 0.803 (0.770-0.837) | 0.612 (0.571-0.653) | 0.680 (0.644-0.716) | 0.752 (0.712-0.793) | 1.188 |
| FSI | 0.862 (0.840-0.883) | 0.001 | 0.756 (0.719-0.792) | 0.808 (0.774-0.841) | 0.724 (0.801-0.767) | 0.836 (0.763-0.728) | -0.823 |
| ZJU | 0.861 (0.839-0.882) | <0.001 | 0.803 (0.770-0.837) | 0.772 (0.736-0.808) | 0.783 (0.749-0.817) | 0.793 (0.758-0.828) | 38.493 |
| TyG | 0.756 (0.727-0.784) | <0.001 | 0.776 (0.741-0.811) | 0.623 (0.582-0.665) | 0.678 (0.642-0.715) | 0.731 (0.690-0.772) | 8.514 |

* *P* values for the difference of AUC between the MPN and other models.

**Table S4** Performance assessment of the MPN and other existing models for the prediction of MAFLD among female participants

| Models | AUC (95% CI) | *P** values | SEN (95% CI) | SPE (95% CI) | PPV (95% CI) | NPV (95% CI) | Cutoff values |
| --- | --- | --- | --- | --- | --- | --- | --- |
| **Female in the training set** |  |  |  |  |  |  |  |
| MPN | 0.859 (0.845-0.873) | - | 0.874 (0.853-0.894) | 0.682 (0.659-0.705) | 0.652 (0.627-0.677) | 0.888 (0.869-0.906) | 0.286 |
| FLI | 0.841 (0.826-0.855) | <0.001 | 0.875 (0.855-0.896) | 0.654 (0.630-0.678) | 0.633 (0.609-0.658) | 0.885 (0.866-0.903) | 42.611 |
| HSI | 0.820 (0.805-0.836) | <0.001 | 0.869 (0.848-0.889) | 0.622 (0.598-0.647) | 0.611 (0.586-0.636) | 0.874 (0.854-0.894) | 36.594 |
| VAI | 0.743 (0.724-0.762) | <0.001 | 0.762 (0.737-0.788) | 0.608 (0.584-0.632) | 0.571 (0.545-0.597) | 0.789 (0.766-0.813) | 1.499 |
| FSI | 0.842 (0.827-0.857) | <0.001 | 0.841 (0.819-0.863) | 0.672 (0.649-0.696) | 0.637 (0.611-0.662) | 0.861 (0.841-0.880) | -1.457 |
| ZJU | 0.829 (0.814-0.845) | <0.001 | 0.866 (0.845-0.887) | 0.636 (0.612-0.660) | 0.619 (0.594-0.644) | 0.874 (0.855-0.894) | 38.950 |
| TyG | 0.748 (0.729-0.767) | <0.001 | 0.717 (0.690-0.745) | 0.649 (0.625-0.673) | 0.583 (0.556-0.610) | 0.771 (0.748-0.794) | 8.496 |
| **Female in the validation set** |  |  |  |  |  |  |  |
| MPN | 0.846 (0.823-0.868) | - | 0.822 (0.787-0.857) | 0.724 (0.690-0.758) | 0.678 (0.639-0.717) | 0.852 (0.822-0.882) | 0.371 |
| FLI | 0.834 (0.811-0.857) | 0.045 | 0.857 (0.825-0.889) | 0.655 (0.618-0.691) | 0.637 (0.599-0.675) | 0.866 (0.836-0.896) | 46.772 |
| HSI | 0.810 (0.785-0.835) | <0.001 | 0.744 (0.704-0.784) | 0.733 (0.699-0.767) | 0.663 (0.623-0.704) | 0.802 (0.770-0.834) | 39.651 |
| VAI | 0.736 (0.707-0.765) | <0.001 | 0.826 (0.792-0.861) | 0.541 (0.503-0.580) | 0.560 (0.523-0.598) | 0.815 (0.779-0.852) | 1.343 |
| FSI | 0.829 (0.806-0.853) | 0.001 | 0.777 (0.739-0.815) | 0.719 (0.685-0.754) | 0.662 (0.622-0.702) | 0.820 (0.788-0.851) | -1.169 |
| ZJU | 0.823 (0.799-0.847) | <0.001 | 0.761 (0.722-0.800) | 0.738 (0.704-0.771) | 0.672 (0.632-0.713) | 0.814 (0.782-0.845) | 41.698 |
| TyG | 0.726 (0.697-0.756) | <0.001 | 0.720 (0.679-0.761) | 0.623 (0.585-0.660) | 0.574 (0.534-0.615) | 0.759 (0.723-0.795) | 8.473 |

* *P* values for the difference of AUC between the MPN and other models.

**Table S5** Performance assessment of the MPN and other existing models for the prediction of MAFLD among participants aged less than 60 years

| Models | AUC (95% CI) | *P** values | SEN (95% CI) | SPE (95% CI) | PPV (95% CI) | NPV (95% CI) | Cutoff values |
| --- | --- | --- | --- | --- | --- | --- | --- |
| **Age<60 in the training set** |  |  |  |  |  |  |  |
| MPN | 0.886 (0.876-0.897) | - | 0.851 (0.833-0.869) | 0.756 (0.737-0.775) | 0.718 (0.696-0.739) | 0.874 (0.859-0.890) | 0.360 |
| FLI | 0.869 (0.857-0.880) | <0.001 | 0.839 (0.820-0.858) | 0.731 (0.712-0.751) | 0.695 (0.673-0.717) | 0.861 (0.845-0.878) | 52.079 |
| HSI | 0.853 (0.840-0.865) | <0.001 | 0.878 (0.861-0.894) | 0.677 (0.657-0.698) | 0.665 (0.643-0.686) | 0.883 (0.867-0.900) | 36.569 |
| VAI | 0.750 (0.734-0.766) | <0.001 | 0.730 (0.707-0.753) | 0.649 (0.628-0.670) | 0.603 (0.580-0.626) | 0.767 (0.747-0.787) | 1.497 |
| FSI | 0.873 (0.861-0.884) | <0.001 | 0.848 (0.830-0.867) | 0.727 (0.707-0.747) | 0.694 (0.672-0.715) | 0.868 (0.852-0.884) | -1.466 |
| ZJU | 0.857 (0.845-0.869) | <0.001 | 0.898 (0.882-0.913) | 0.664 (0.643-0.685) | 0.661 (0.640-0.682) | 0.899 (0.883-0.914) | 37.763 |
| TyG | 0.755 (0.738-0.771) | <0.001 | 0.714 (0.691-0.737) | 0.661 (0.640-0.682) | 0.606 (0.582-0.629) | 0.760 (0.740-0.780) | 8.497 |
| **Age<60 in the validation set** |  |  |  |  |  |  |  |
| MPN | 0.880 (0.863-0.897) | - | 0.848 (0.820-0.877) | 0.756 (0.726-0.785) | 0.725 (0.692-0.757) | 0.868 (0.843-0.893) | 0.368 |
| FLI | 0.865 (0.847-0.883) | 0.001 | 0.850 (0.822-0.878) | 0.703 (0.671-0.734) | 0.684 (0.652-0.717) | 0.861 (0.835-0.887) | 50.754 |
| HSI | 0.846 (0.827-0.866) | <0.001 | 0.799 (0.768-0.830) | 0.733 (0.703-0.763) | 0.694 (0.660-0.728) | 0.828 (0.801-0.855) | 38.581 |
| VAI | 0.753 (0.728-0.778) | <0.001 | 0.759 (0.726-0.793) | 0.637 (0.604-0.670) | 0.613 (0.579-0.648) | 0.777 (0.746-0.809) | 1.393 |
| FSI | 0.868 (0.850-0.886) | 0.002 | 0.746 (0.712-0.780) | 0.808 (0.781-0.835) | 0.746 (0.712-0.780) | 0.808 (0.781-0.835) | -1.046 |
| ZJU | 0.854 (0.835-0.873) | <0.001 | 0.866 (0.839-0.893) | 0.688 (0.656-0.720) | 0.678 (0.646-0.710) | 0.871 (0.846-0.897) | 38.551 |
| TyG | 0.754 (0.729-0.779) | <0.001 | 0.708 (0.673-0.744) | 0.684 (0.653-0.716) | 0.630 (0.594-0.665) | 0.756 (0.725-0.786) | 8.515 |

* *P* values for the difference of AUC between the MPN and other models.

**Table S6** Performance assessment of the MPN and other existing models for the prediction of MAFLD among participants aged greater than or equal to 60 years

| Models | AUC (95% CI) | *P** values | SEN (95% CI) | SPE (95% CI) | PPV (95% CI) | NPV (95% CI) | Cutoff values |
| --- | --- | --- | --- | --- | --- | --- | --- |
| **Age≥60 in the training set** |  |  |  |  |  |  |  |
| MPN | 0.823 (0.803-0.842) | - | 0.738 (0.710-0.767) | 0.744 (0.715-0.774) | 0.758 (0.730-0.787) | 0.724 (0.694-0.754) | 0.537 |
| FLI | 0.803 (0.782-0.823) | <0.001 | 0.711 (0.682-0.741) | 0.741 (0.711-0.771) | 0.749 (0.719-0.778) | 0.703 (0.673-0.734) | 64.216 |
| HSI | 0.777 (0.755-0.798) | <0.001 | 0.734 (0.705-0.763) | 0.694 (0.663-0.726) | 0.723 (0.693-0.752) | 0.706 (0.675-0.738) | 37.350 |
| VAI | 0.689 (0.664-0.714) | <0.001 | 0.703 (0.673-0.734) | 0.603 (0.569-0.636) | 0.658 (0.627-0.688) | 0.652 (0.618-0.686) | 1.541 |
| FSI | 0.788 (0.766-0.809) | <0.001 | 0.772 (0.745-0.800) | 0.656 (0.624-0.689) | 0.709 (0.680-0.738) | 0.727 (0.695-0.759) | -0.935 |
| ZJU | 0.785 (0.763-0.806) | <0.001 | 0.770 (0.742-0.798) | 0.663 (0.630-0.695) | 0.712 (0.684-0.741) | 0.727 (0.695-0.759) | 39.001 |
| TyG | 0.705 (0.681-0.730) | <0.001 | 0.700 (0.670-0.730) | 0.615 (0.582-0.648) | 0.663 (0.633-0.694) | 0.654 (0.620-0.688) | 8.640 |
| **Age≥60 in the validation set** |  |  |  |  |  |  |  |
| MPN | 0.823 (0.793-0.852) | - | 0.762 (0.719-0.805) | 0.733 (0.687-0.779) | 0.752 (0.709-0.795) | 0.744 (0.698-0.789) | 0.510 |
| FLI | 0.806 (0.775-0.838) | 0.064 | 0.870 (0.837-0.904) | 0.604 (0.553-0.655) | 0.700 (0.659-0.741) | 0.814 (0.767-0.861) | 49.310 |
| HSI | 0.766 (0.732-0.800) | <0.001 | 0.672 (0.625-0.719) | 0.758 (0.714-0.803) | 0.747 (0.701-0.793) | 0.685 (0.639-0.731) | 38.507 |
| VAI | 0.710 (0.672-0.747) | <0.001 | 0.775 (0.733-0.817) | 0.567 (0.516-0.619) | 0.655 (0.611-0.700) | 0.704 (0.651-0.757) | 1.346 |
| FSI | 0.796 (0.764-0.828) | <0.001 | 0.733 (0.688-0.777) | 0.733 (0.687-0.779) | 0.745 (0.700-0.789) | 0.721 (0.675-0.767) | -0.630 |
| ZJU | 0.774 (0.740-0.807) | <0.001 | 0.796 (0.756-0.837) | 0.643 (0.593-0.693) | 0.703 (0.660-0.747) | 0.748 (0.700-0.797) | 38.507 |
| TyG | 0.714 (0.677-0.751) | <0.001 | 0.574 (0.524-0.624) | 0.756 (0.711-0.800) | 0.714 (0.663-0.765) | 0.626 (0.580-0.671) | 8.863 |

* *P* values for the difference of AUC between the MPN and other models.

**Table S7** Performance assessment of the MPN and other existing models for the prediction of MAFLD in participants without hypertension

| Models | AUC (95% CI) | *P** values | SEN (95% CI) | SPE (95% CI) | PPV (95% CI) | NPV (95% CI) | Cutoff values |
| --- | --- | --- | --- | --- | --- | --- | --- |
| **Non-hypertension in the training set** |  |  |  |  |  |  |  |
| MPN | 0.880 (0.868-0.891) | - | 0.850 (0.830-0.869) | 0.750 (0.731-0.769) | 0.674 (0.650-0.697) | 0.891 (0.877-0.906) | 0.323 |
| FLI | 0.862 (0.849-0.874) | <0.001 | 0.852 (0.832-0.872) | 0.719 (0.700-0.739) | 0.648 (0.625-0.671) | 0.889 (0.874-0.904) | 46.104 |
| HSI | 0.839 (0.825-0.852) | <0.001 | 0.856 (0.836-0.876) | 0.683 (0.663-0.703) | 0.621 (0.598-0.644) | 0.887 (0.871-0.902) | 36.035 |
| VAI | 0.746 (0.729-0.763) | <0.001 | 0.696 (0.670-0.721) | 0.674 (0.654-0.695) | 0.565 (0.540-0.590) | 0.785 (0.766-0.804) | 1.541 |
| FSI | 0.865 (0.853-0.877) | <0.001 | 0.859 (0.840-0.879) | 0.700 (0.680-0.720) | 0.635 (0.612-0.658) | 0.891 (0.876-0.907) | -1.786 |
| ZJU | 0.847 (0.835-0.860) | <0.001 | 0.886 (0.868-0.904) | 0.661 (0.641-0.682) | 0.613 (0.591-0.636) | 0.905 (0.890-0.920) | 37.183 |
| TyG | 0.751 (0.735-0.768) | <0.001 | 0.731 (0.706-0.755) | 0.641 (0.621-0.662) | 0.553 (0.529-0.577) | 0.797 (0.777-0.816) | 8.482 |
| **Non-hypertension in the validation set** |  |  |  |  |  |  |  |
| MPN | 0.874 (0.856-0.892) | - | 0.818 (0.785-0.850) | 0.773 (0.744-0.801) | 0.694 (0.658-0.729) | 0.871 (0.847-0.894) | 0.372 |
| FLI | 0.860 (0.841-0.879) | 0.009 | 0.883 (0.855-0.910) | 0.685 (0.653-0.716) | 0.638 (0.603-0.673) | 0.903 (0.880-0.925) | 44.197 |
| HSI | 0.835 (0.815-0.856) | <0.001 | 0.804 (0.771-0.838) | 0.710 (0.680-0.741) | 0.636 (0.600-0.672) | 0.852 (0.826-0.878) | 37.037 |
| VAI | 0.758 (0.732-0.783) | <0.001 | 0.786 (0.751-0.821) | 0.624 (0.591-0.656) | 0.568 (0.532-0.604) | 0.822 (0.793-0.852) | 1.305 |
| FSI | 0.864 (0.846-0.883) | 0.024 | 0.879 (0.851-0.907) | 0.675 (0.644-0.707) | 0.630 (0.596-0.665) | 0.899 (0.875-0.922) | -1.871 |
| ZJU | 0.849 (0.829-0.868) | <0.001 | 0.821 (0.789-0.854) | 0.722 (0.692-0.752) | 0.650 (0.615-0.686) | 0.865 (0.840-0.890) | 38.551 |
| TyG | 0.752 (0.726-0.778) | <0.001 | 0.724 (0.687-0.762) | 0.673 (0.641-0.704) | 0.582 (0.545-0.620) | 0.795 (0.766-0.824) | 8.492 |

* *P* values for the difference of AUC between the MPN and other models.

**Table S8** Performance assessment of the MPN and other existing models for the prediction of MAFLD in participants with hypertension

| Models | AUC (95% CI) | *P** values | SEN (95% CI) | SPE (95% CI) | PPV (95% CI) | NPV (95% CI) | Cutoff values |
| --- | --- | --- | --- | --- | --- | --- | --- |
| **Hypertension in the training set** |  |  |  |  |  |  |  |
| MPN | 0.823 (0.804-0.842) | - | 0.688 (0.660-0.715) | 0.793 (0.764-0.822) | 0.827 (0.803-0.852) | 0.637 (0.606-0.668) | 0.641 |
| FLI | 0.801 (0.780-0.821) | <0.001 | 0.728 (0.701-0.754) | 0.727 (0.696-0.759) | 0.794 (0.769-0.819) | 0.649 (0.617-0.681) | 71.777 |
| HSI | 0.783 (0.762-0.805) | <0.001 | 0.722 (0.696-0.749) | 0.727 (0.696-0.759) | 0.793 (0.768-0.818) | 0.644 (0.612-0.676) | 39.483 |
| VAI | 0.687 (0.663-0.712) | <0.001 | 0.707 (0.680-0.734) | 0.594 (0.559-0.630) | 0.716 (0.689-0.743) | 0.584 (0.549-0.618) | 1.603 |
| FSI | 0.796 (0.775-0.816) | <0.001 | 0.776 (0.751-0.801) | 0.668 (0.634-0.701) | 0.771 (0.747-0.796) | 0.673 (0.639-0.707) | -0.420 |
| ZJU | 0.787 (0.766-0.809) | <0.001 | 0.783 (0.759-0.808) | 0.674 (0.641-0.708) | 0.777 (0.752-0.801) | 0.682 (0.649-0.716) | 40.231 |
| TyG | 0.695 (0.670-0.719) | <0.001 | 0.705 (0.678-0.732) | 0.594 (0.559-0.630) | 0.715 (0.688-0.742) | 0.582 (0.547-0.617) | 8.630 |
| **Hypertension in the validation set** |  |  |  |  |  |  |  |
| MPN | 0.823 (0.793-0.852) | - | 0.718 (0.677-0.759) | 0.803 (0.760-0.846) | 0.838 (0.802-0.874) | 0.668 (0.621-0.714) | 0.620 |
| FLI | 0.803 (0.772-0.834) | 0.016 | 0.726 (0.686-0.767) | 0.739 (0.692-0.787) | 0.798 (0.760-0.836) | 0.656 (0.608-0.704) | 72.172 |
| HSI | 0.768 (0.735-0.801) | <0.001 | 0.746 (0.706-0.785) | 0.676 (0.625-0.726) | 0.765 (0.726-0.804) | 0.652 (0.602-0.703) | 39.212 |
| VAI | 0.690 (0.653-0.728) | <0.001 | 0.654 (0.611-0.697) | 0.648 (0.597-0.700) | 0.725 (0.683-0.768) | 0.569 (0.519-0.619) | 1.759 |
| FSI | 0.802 (0.771-0.832) | 0.002 | 0.816 (0.781-0.851) | 0.661 (0.610-0.712) | 0.773 (0.736-0.810) | 0.717 (0.666-0.768) | -0.502 |
| ZJU | 0.772 (0.739-0.805) | <0.001 | 0.748 (0.709-0.787) | 0.697 (0.647-0.747) | 0.778 (0.739-0.816) | 0.661 (0.611-0.711) | 41.316 |
| TyG | 0.699 (0.662-0.736) | <0.001 | 0.551 (0.506-0.596) | 0.745 (0.698-0.792) | 0.754 (0.709-0.800) | 0.539 (0.494-0.585) | 8.860 |

* *P* values for the difference of AUC between the MPN and other models.

**Table S9** Performance assessment of the MPN and other existing models for the prediction of MAFLD in participants without diabetes

| Models | AUC (95% CI) | *P** values | SEN (95% CI) | SPE (95% CI) | PPV (95% CI) | NPV (95% CI) | Cutoff values |
| --- | --- | --- | --- | --- | --- | --- | --- |
| **Non-diabetes in the training set** |  |  |  |  |  |  |  |
| MPN | 0.864 (0.853-0.875) | - | 0.850 (0.832-0.867) | 0.712 (0.694-0.729) | 0.655 (0.635-0.676) | 0.880 (0.866-0.894) | 0.319 |
| FLI | 0.852 (0.841-0.863) | <0.001 | 0.853 (0.836-0.870) | 0.690 (0.672-0.708) | 0.640 (0.619-0.660) | 0.879 (0.865-0.894) | 46.425 |
| HSI | 0.821 (0.808-0.833) | <0.001 | 0.829 (0.811-0.848) | 0.668 (0.649-0.686) | 0.617 (0.597-0.637) | 0.858 (0.843-0.874) | 36.035 |
| VAI | 0.722 (0.706-0.737) | <0.001 | 0.674 (0.652-0.697) | 0.662 (0.643-0.680) | 0.563 (0.541-0.585) | 0.759 (0.741-0.777) | 1.541 |
| FSI | 0.845 (0.833-0.856) | <0.001 | 0.858 (0.841-0.875) | 0.656 (0.637-0.674) | 0.617 (0.597-0.637) | 0.878 (0.863-0.892) | -1.691 |
| ZJU | 0.831 (0.819-0.843) | <0.001 | 0.840 (0.822-0.858) | 0.676 (0.657-0.694) | 0.626 (0.605-0.646) | 0.867 (0.852-0.882) | 37.763 |
| TyG | 0.722 (0.706-0.737) | <0.001 | 0.773 (0.752-0.793) | 0.559 (0.540-0.579) | 0.531 (0.511-0.551) | 0.792 (0.773-0.811) | 8.375 |
| **Non-diabetes in the validation set** |  |  |  |  |  |  |  |
| MPN | 0.861 (0.844-0.878) | - | 0.818 (0.790-0.847) | 0.748 (0.722-0.774) | 0.682 (0.650-0.713) | 0.862 (0.840-0.884) | 0.368 |
| FLI | 0.849 (0.831-0.866) | 0.009 | 0.878 (0.854-0.903) | 0.654 (0.625-0.683) | 0.626 (0.596-0.656) | 0.891 (0.869-0.913) | 44.197 |
| HSI | 0.814 (0.795-0.834) | <0.001 | 0.751 (0.719-0.783) | 0.730 (0.703-0.756) | 0.647 (0.614-0.679) | 0.816 (0.792-0.841) | 37.668 |
| VAI | 0.730 (0.706-0.754) | <0.001 | 0.763 (0.731-0.794) | 0.601 (0.572-0.631) | 0.558 (0.526-0.589) | 0.794 (0.766-0.822) | 1.305 |
| FSI | 0.842 (0.825-0.860) | <0.001 | 0.854 (0.828-0.880) | 0.648 (0.619-0.676) | 0.615 (0.584-0.645) | 0.871 (0.847-0.894) | -1.653 |
| ZJU | 0.825 (0.806-0.844) | <0.001 | 0.801 (0.772-0.831) | 0.705 (0.678-0.732) | 0.641 (0.610-0.673) | 0.843 (0.819-0.867) | 38.511 |
| TyG | 0.724 (0.700-0.748) | <0.001 | 0.685 (0.651-0.720) | 0.659 (0.630-0.687) | 0.570 (0.536-0.603) | 0.761 (0.733-0.788) | 8.485 |

* *P* values for the difference of AUC between the MPN and other models.

**Table S10** Performance assessment of the MPN and other existing models for the prediction of MAFLD in participants with diabetes

| Models | AUC (95% CI) | *P** values | SEN (95% CI) | SPE (95% CI) | PPV (95% CI) | NPV (95% CI) | Cutoff values |
| --- | --- | --- | --- | --- | --- | --- | --- |
| **Diabetes in the training set** |  |  |  |  |  |  |  |
| MPN | 0.803 (0.773-0.833) | - | 0.749 (0.717-0.781) | 0.698 (0.644-0.752) | 0.864 (0.836-0.891) | 0.522 (0.471-0.573) | 0.688 |
| FLI | 0.760 (0.727-0.794) | <0.001 | 0.749 (0.717-0.781) | 0.636 (0.580-0.693) | 0.840 (0.812-0.869) | 0.499 (0.446-0.551) | 71.288 |
| HSI | 0.757 (0.723-0.791) | <0.001 | 0.789 (0.759-0.819) | 0.600 (0.542-0.658) | 0.834 (0.806-0.863) | 0.527 (0.472-0.582) | 39.646 |
| VAI | 0.684 (0.646-0.722) | <0.001 | 0.725 (0.692-0.758) | 0.582 (0.524-0.640) | 0.816 (0.785-0.846) | 0.453 (0.401-0.505) | 1.756 |
| FSI | 0.762 (0.729-0.795) | <0.001 | 0.694 (0.660-0.728) | 0.724 (0.671-0.776) | 0.865 (0.837-0.893) | 0.481 (0.433-0.529) | 0.331 |
| ZJU | 0.753 (0.719-0.788) | <0.001 | 0.768 (0.737-0.799) | 0.611 (0.553-0.669) | 0.834 (0.806-0.863) | 0.508 (0.454-0.561) | 41.935 |
| TyG | 0.680 (0.642-0.717) | <0.001 | 0.738 (0.705-0.770) | 0.538 (0.479-0.597) | 0.803 (0.772-0.834) | 0.446 (0.392-0.499) | 8.807 |
| **Diabetes in the validation set** |  |  |  |  |  |  |  |
| MPN | 0.785 (0.736-0.833) | - | 0.814 (0.770-0.857) | 0.639 (0.554-0.725) | 0.850 (0.809-0.891) | 0.578 (0.494-0.661) | 0.640 |
| FLI | 0.764 (0.713-0.815) | 0.117 | 0.748 (0.700-0.797) | 0.639 (0.554-0.725) | 0.839 (0.795-0.882) | 0.503 (0.425-0.582) | 72.196 |
| HSI | 0.738 (0.686-0.790) | 0.003 | 0.824 (0.781-0.866) | 0.525 (0.436-0.613) | 0.813 (0.769-0.856) | 0.542 (0.452-0.632) | 39.115 |
| VAI | 0.688 (0.631-0.745) | <0.001 | 0.676 (0.624-0.729) | 0.631 (0.546-0.717) | 0.821 (0.774-0.869) | 0.438 (0.364-0.511) | 1.934 |
| FSI | 0.762 (0.712-0.813) | 0.101 | 0.598 (0.543-0.653) | 0.803 (0.733-0.874) | 0.884 (0.840-0.928) | 0.443 (0.378-0.509) | 0.798 |
| ZJU | 0.742 (0.688-0.769) | 0.007 | 0.804 (0.759-0.848) | 0.574 (0.486-0.662) | 0.826 (0.782-0.869) | 0.538 (0.453-0.624) | 42.158 |
| TyG | 0.672 (0.616-0.729) | <0.001 | 0.693 (0.641-0.745) | 0.590 (0.503-0.677) | 0.809 (0.762-0.857) | 0.434 (0.358-0.509) | 8.939 |

* *P* values for the difference of AUC between the MPN and other models.
